# Endogenous CCL2 neutralization restricts HIV-1 replication in primary human macrophages by inhibiting viral DNA accumulation

**DOI:** 10.1186/s12977-014-0132-6

**Published:** 2015-01-22

**Authors:** Michela Sabbatucci, Daniela Angela Covino, Cristina Purificato, Alessandra Mallano, Maurizio Federico, Jing Lu, Arturo Ottavio Rinaldi, Matteo Pellegrini, Roberta Bona, Zuleika Michelini, Andrea Cara, Stefano Vella, Sandra Gessani, Mauro Andreotti, Laura Fantuzzi

**Affiliations:** Department of Hematology, Oncology and Molecular Medicine, Istituto Superiore di Sanità, Rome, Italy; Department of Therapeutic Research and Medicines Evaluation, Istituto Superiore di Sanità, Rome, Italy; National AIDS Center, Istituto Superiore di Sanità, Rome, Italy; Department of Molecular, Cell, and Developmental Biology, University of California Los Angeles, Los Angeles, CA 90095 USA

**Keywords:** Monocyte-derived macrophage, CCL2, HIV-1, Restriction, SAMHD1, APOBEC3A

## Abstract

**Background:**

Macrophages are key targets of HIV-1 infection. We have previously described that the expression of CC chemokine ligand 2 (CCL2) increases during monocyte differentiation to macrophages and it is further up-modulated by HIV-1 exposure. Moreover, CCL2 acts as an autocrine factor that promotes viral replication in infected macrophages. In this study, we dissected the molecular mechanisms by which CCL2 neutralization inhibits HIV-1 replication in monocyte-derived macrophages (MDM), and the potential involvement of the innate restriction factors protein sterile alpha motif (SAM) histidine/aspartic acid (HD) domain containing 1 (SAMHD1) and apolipoprotein B mRNA-editing, enzyme-catalytic, polypeptide-like 3 (APOBEC3) family members.

**Results:**

CCL2 neutralization potently reduced the number of p24 Gag^+^ cells during the course of either productive or single cycle infection with HIV-1. In contrast, CCL2 blocking did not modify entry of HIV-1 based Virus Like Particles, thus demonstrating that the restriction involves post-entry steps of the viral life cycle. Notably, the accumulation of viral DNA, both total, integrated and 2-LTR circles, was strongly impaired by neutralization of CCL2. Looking for correlates of HIV-1 DNA accumulation inhibition, we found that the antiviral effect of CCL2 neutralization was independent of the modulation of SAMHD1 expression or function. Conversely, a strong and selective induction of APOBEC3A expression, to levels comparable to those of freshly isolated monocytes, was associated with the inhibition of HIV-1 replication mediated by CCL2 blocking. Interestingly, the CCL2 neutralization mediated increase of APOBEC3A expression was type I IFN independent. Moreover, the transcriptome analysis of the effect of CCL2 blocking on global gene expression revealed that the neutralization of this chemokine resulted in the upmodulation of additional genes involved in the defence response to viruses.

**Conclusions:**

Neutralization of endogenous CCL2 determines a profound restriction of HIV-1 replication in primary MDM affecting post-entry steps of the viral life cycle with a mechanism independent of SAMHD1. In addition, CCL2 blocking is associated with induction of APOBEC3A expression, thus unravelling a novel mechanism which might contribute to regulate the expression of innate intracellular viral antagonists *in vivo*. Thus, our study may potentially lead to the development of new therapeutic strategies for enhancing innate cellular defences against HIV-1 and protecting macrophages from infection.

**Electronic supplementary material:**

The online version of this article (doi:10.1186/s12977-014-0132-6) contains supplementary material, which is available to authorized users.

## Background

Early after its discovery, it has been established that HIV-1, like other retroviruses, infects not only CD4^+^ T lymphocytes but also macrophages. The presence of HIV-1-infected macrophages *in vivo* has been documented in various tissues, including brain, lung and gut [1-10]. Although their precise contribution to the infection and pathogenesis of HIV-1 is still a matter of debate, the importance of macrophages in these processes is highlighted by their involvement in early-stage viral transmission, persistence, and virus dissemination throughout the body of the host [11,12]. Once infected, macrophages promote rapid virus dissemination by transmitting viral particles to CD4^+^ T cells via a transit “virological synapse” [13]. As macrophage has the ability to cross the blood-tissue barrier and to migrate into tissues, HIV-infected macrophages are potent agents for viral delivery to all tissues and organs. Macrophages are considered as viral reservoirs because they are long-lived cells resistant to the cytopathic effects of HIV-1 and “hide” the virus in safe intracellular compartments [14]. This allows maintaining a hidden HIV-1 reservoir for ongoing infection, hardly eradicable by currently available pharmacological therapies [15]. Therefore, efforts directed to defining the mechanisms and factors controlling HIV-1 replication in macrophages may provide the basis for devising new, long-term successful treatment of infected individuals [11].

Chemokines and their receptors are deeply involved in the control of HIV-1 infection [16]. In addition to CCR5- and CXCR4-binding chemokines interfering with HIV-1 infection at the entry level, other chemokines have been shown to play a role in this infection [17]. In particular, CC chemokine ligand 2 (CCL2; formerly monocyte chemotactic protein-1, MCP-1) is induced during several human acute and chronic viral infections [18,19]. In addition to HIV-1 infection [20,21], virus-derived proteins such as gp120 [22], Nef [23], matrix protein p17 [24] and transactivator protein Tat [25,26] increase the expression and release of this chemokine. CCL2 is produced by a variety of cell types, with monocytes/macrophages representing the major source among leukocytes [18,19]. Although the precise contribution of CCL2 in HIV-1 infection and pathogenesis remains to be established, growing evidence suggests that it may play important roles in these processes [18]. We previously found that CCL2 is up-regulated during monocyte differentiation to macrophages and it is further increased upon HIV-1 infection or exposure to viral proteins. Furthermore, this chemokine acts as an autocrine factor that sustains viral replication in HIV-1 infected cells [21]. However, the mechanism(s) by which CCL2 fosters HIV-1 production remains to be elucidated.

A variety of host cell factors can interfere with HIV-1 replication [27-29]. Among these, the protein sterile alpha motif (SAM) histidine/aspartic acid (HD) domain containing 1 (SAMHD1) was recently identified as a restriction factor in myeloid cells [30,31]. SAMHD1 is a dGTP-regulated deoxynucleotide triphosphates (dNTP) hydrolase that limits the pool of dNTP available for reverse transcription, therefore reducing HIV-1 infection of myeloid cells [32-34]. Recently, it has been shown that SAMHD1 can restrict HIV-1 infection also through degradation of viral RNA [35]. In addition to SAMHD1, members of the apolipoprotein B mRNA-editing, enzyme-catalytic, polypeptide-like 3 (APOBEC3; A3) family of cytidine deaminases are potent innate intracellular viral antagonists which restrict HIV-1 replication in target cells [36-38]. The human genome encodes seven A3 genes (A3A, A3B, A3C, A3D/E, A3F, A3G and A3H) [39]. A3 proteins are widely expressed in hematopoietic cell populations, including T cells, B cells and myeloid cells [40,41]. In particular, A3A and A3G are critical for monocyte resistance to infection and their decreased expression during macrophage differentiation results in a permissive target cell population [42]. Beyond the well known capacity of type I interferon to induce the expression of some A3 family members [43], little is known concerning the factors and mechanisms regulating the expression of these enzymes in myeloid cells. Thus, their identification might offer great opportunities to prevent HIV-1 from infecting and persisting in macrophages.

In this study, we aimed at dissecting the molecular mechanisms by which CCL2 blocking restricts HIV-1 replication in macrophages. We report that neutralization of this chemokine reduces the number of infected macrophages, without affecting viral entry, and impairs viral DNA accumulation. Interestingly, whereas SAMHD1 expression and function are not altered by CCL2 blocking, a strong and selective induction of A3A expression is associated with the CCL2 neutralization-mediated restriction of HIV-1 replication. Therefore, our study highlights the potential of targeting this chemokine to impair early steps of HIV-1 replication and to strengthen innate intracellular pathways to thwart HIV-1 infection.

## Results

### Neutralization of endogenous CCL2 reduces the proportion of HIV-1 infected MDM

**Figure 1:**
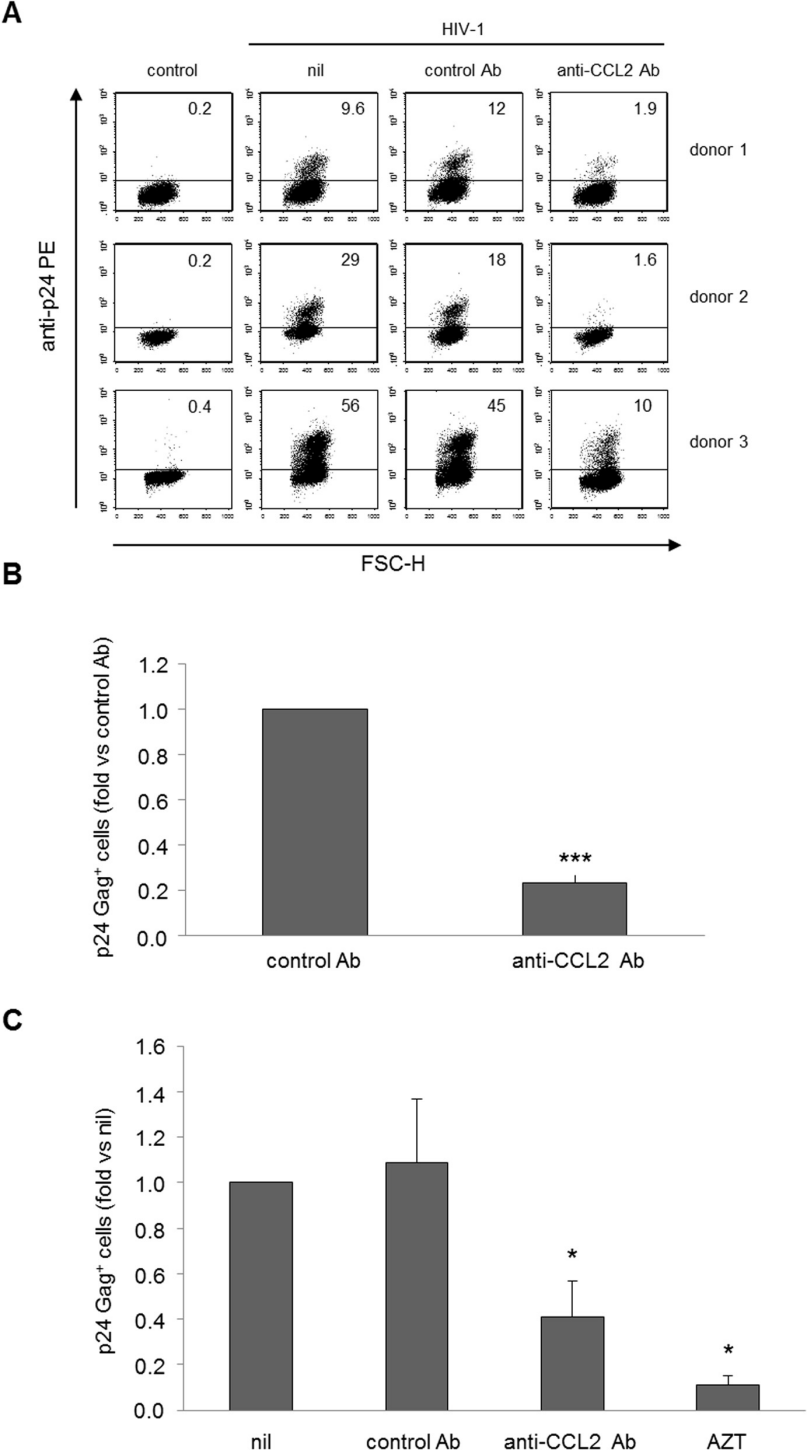
**Neutralization of endogenous CCL2 reduces the proportion of HIV-1 infected MDM.** MDM were treated with anti-CCL2 or control Ab (2.5 μg/ml) for 20 h. **(A, B)** Cells were then infected with HIV-1_BaL_ (3000 TCID_50_ per well) as described in Methods. After 14 days, cells were recovered and HIV-1 Gag expression was evaluated by flow cytometry. Values indicate the percentage of p24 Gag^+^ cells for each sample. In A, the results from 3 representative donors out of 22 tested are shown. In B, data represent mean values (+SE) of the results obtained with all the donors analyzed. ***p < 0.001 (anti-CCL2 Ab *vs.* control Ab). **(C)** Cells were challenged with (VSV-G) HIV-1 (250 ng CAp24 equivalent per 10^5^ cells) in a single-round infectivity assay. Some cultures were also treated with AZT (10 μM). The percentage of p24 Gag^+^ cells was assessed by flow cytometry 3 days after infection. Data represent mean values (+SE) of the results obtained with MDM from 4 different donors. *p < 0.05 (anti-CCL2 Ab and AZT *vs.* nil).

In our previous studies we found that CCL2 neutralization reduced the release of p24 Gag antigen in HIV-1 infected monocyte-derived macrophages (MDM) [21]. To examine the mechanisms by which CCL2 blocking inhibits HIV-1 replication in these cells, experiments were performed to assess whether neutralization of this chemokine affected the total number of infected cells. Toward this aim, MDM cultures established from several donors were exposed for 20 h to anti-CCL2 or control antibody (Ab) and then challenged with HIV-1_BaL_. The proportion of infected cells was determined 14 days post-infection by flow cytometry after permeabilization and staining for intracellular p24 Gag. A marked variability in the percentage of p24 Gag^+^ cells was observed among donors, ranging from 2.4 to 77.3% [median value: 28.0 ± 5.4 (SE); n = 22]. Despite this variability, treatment with anti-CCL2 Ab strongly reduced the percentage of p24 Gag^+^ cells in all the donors analyzed (Figure 1A). In spite of an occasional effect of control Ab on this parameter, anti-CCL2 Ab significantly reduced the proportion of p24 Gag^+^ cells with respect to control Ab treatment in all the donors analyzed. In particular, as shown in Figure 1B, exposure to anti-CCL2 Ab reduced the fraction of p24 Gag^+^ cells to 0.23 ± 0.04 (SE) fold compared to control Ab treatment (p < 0.001). In keeping with previous results [21], the reduction of infected cells was associated with a marked inhibition of the release of p24 Gag in culture medium of MDM treated with anti-CCL2 Ab [0.19 ± 0.05 (SE) fold *vs.* control Ab; p < 0.001]. Furthermore, an increase of CCL2 production following HIV-1 infection was found in these donors. In particular, at 14 days post-infection the median CCL2 secretion in uninfected cells was 8,410.9 ± 1,027.5 (SE) pg/ml, whereas it increased to 18,982.9 ± 2,102.1 (SE) pg/ml (p < 0.001) upon infection with HIV-1. Measurement of CCL2 release in Ab treated cultures showed that the anti-CCL2 Ab effectively neutralized the chemokine produced by MDM (Additional file 1: Figure S1A).

We then investigated whether CCL2 blocking also inhibited a single cycle HIV-1 infection. To this aim, we used a recombinant virus, mutated in the e*nv* gene and pseudotyped with Vesicular Stomatitis Virus G-protein (VSV-G), which enters macrophages through a CD4/CCR5-independent pathway and completes only a single round of infection [44]. MDM exposed for 20 h to anti-CCL2 or control Ab were challenged with (VSV-G) HIV-1 and the percentage of p24 Gag^+^ cells was measured 3 days post-infection. The percentage of p24 Gag^+^ cells ranged from 17.1 to 77.5% [median value: 49.4 ± 24.7 (SE); n = 4]. Despite this variability, treatment with anti-CCL2 Ab markedly reduced the percentage of p24 Gag^+^ MDM in all the donors analyzed (Figure 1C). In particular, the proportion of p24 Gag^+^ cells following treatment with anti-CCL2 Ab was reduced to 0.41 ± 0.16 (SE) fold with respect to untreated cells (p < 0.05). Control experiments were performed in the presence of the reverse-transcriptase inhibitor zidovudine (AZT) to ensure that the detected fraction of infected cells was not biased by cell membrane-attached p24 Gag. As expected, a strong reduction in the fraction of p24 Gag^+^ cells was observed in the presence of AZT. Measurement of CCL2 release in Ab treated cultures showed that the anti-CCL2 Ab effectively neutralized the chemokine produced by MDM also in these conditions (Additional file 1: Figure S1B).

### Endogenous CCL2 neutralization does not affect entry of HIV-1 in MDM

**Figure 2:**
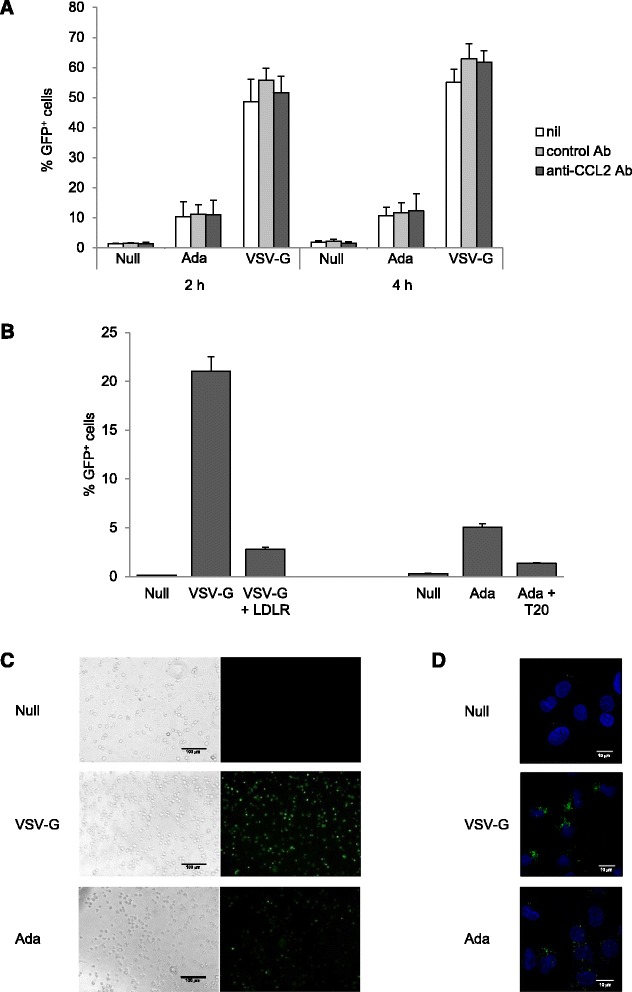
**Endogenous CCL2 neutralization does not affect HIV-1 entry in MDM. (A)** MDM were treated with anti-CCL2 or control Ab (2.5 μg/ml) for 20 h and then challenged with Null-VLPs, VSV-G-VLPs or Ada-VLPs (1 μg of CAp24 equivalent per 10^5^ cells) as described in Methods. After 2 and 4 h, the percentage of GFP^+^ cells was assessed by flow cytometry. Data represent mean values (+SE) of the results obtained with MDM from 4 different donors. **(B)** MDM were treated as in A and then challenged with VSV-G-VLPs or Ada-VLPs either in the presence or in the absence of soluble LDLR (5 μg/ml) or T20 (1 μg/ml). The results obtained with 1 out of 2 different donors analyzed are shown. **(C)** Fluorescence microscope analysis of MDM 2 hours after the challenge with Null-VLPs, VSV-G-VLPs or Ada-VLPs. Phase-contrast micrographs of the same fields are shown on the left. Bars mark 100 μm. **(D)** Confocal microscope analysis of MDM 2 hours after the challenge with Null-VLPs, VSV-G-VLPs or Ada-VLPs. Images represent a Z-projection of 5 optical sections taken near the middle of the cell nucleus. VLP signal is shown in green, while nuclei are stained with DAPI (blue). Bars mark 10 μm.

We then investigated whether CCL2 blocking-mediated inhibition of HIV-1 replication could be due to reduced viral entry in MDM. To this aim, we used fluorescent HIV-1-based Viral Like Particles (VLPs) pseudotyped with either VSV-G or R5 HIV-1 Env from the Ada strain (VSV-G-VLPs and Ada-VLPs, respectively), which has been previously shown to enter different types of cells with high efficiency [45,46]. MDM exposed for 20 h to anti-CCL2 or control Ab were challenged with VLPs and the percentage of GFP^+^ cells was measured after 2 and 4 h of incubation at 37°C. Control experiments were performed by incubating cells at 4°C. As shown in Figure 2A, about 50% of MDM were GFP^+^ following 2 h of incubation with VSV-G-VLPs, whereas viral entry driven by Ada-VLPs was less efficient and rendered fluorescent about 10% of the cells. Similar proportions of GFP^+^ cells were detected following 4 h of incubation at 37°C. Interestingly, treatment with anti-CCL2 or control Ab did not modify entry of either Ada-VLPs or VSV-G-VLPs (Figure 2A and Additional file 2: Figure S2). Control experiments were performed in the presence of soluble LDLR or T20 to block fusion of VSV-G-VLPs or Ada-VLPs, respectively [47,48]. As expected, a strong reduction in the fraction of GFP^+^ cells was observed in these conditions, thus confirming that the fluorescence detected in target cells indeed relied on authentic viral fusion events (Figure 2B). Microscopic analysis of target cells showed a strong fluorescence associated with VSV-G-VLPs challenged MDM, while a significant lower signal was detected in the cells challenged with Ada-VLPs (Figure 2C). Finally, the internalization of VLPs was further confirmed by confocal microscope analysis (Figure 2D).

To verify that anti-CCL2 Ab did not affect reverse transcriptase (RT) activity and early post-entry events, we evaluated HIV-1 DNA synthesis by semi-quantitative PCR assay at 24 h post-infection. Primers were used as described to amplify different regions of the HIV genome and to estimate the extent of reverse transcription at three replicative steps that occur in subsequent order during reverse transcription: R-U5, initial minus strand synthesis; R-PB, initial plus-strand synthesis up to the PB region; and R-gag, complete minus-strand synthesis [49]. Results indicate that the presence of anti-CCL2 Ab did not influence HIV-1 DNA synthesis in the infected cells early post-infection (Additional file 3: Figure S3A). These results were confirmed by further evaluation of the HIV-1 DNA intermediates by a quantitative PCR Real Time assay (Additional file 3: Figure S3B).

These results clearly demonstrate that CCL2 blocking did not show any interference in both entry and RT activity.

### Neutralization of endogenous CCL2 impairs HIV-1 DNA accumulation in MDM

**Figure 3:**
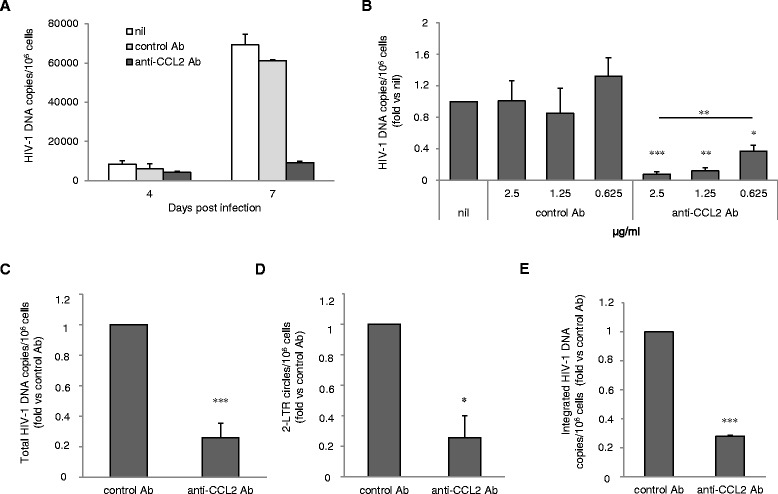
**Neutralization of endogenous CCL2 impairs HIV-1 DNA accumulation in MDM. (A)** MDM were treated with anti-CCL2 or control Ab (2.5 μg/ml) for 20 h and then infected with HIV-1_BaL_ (3000 TCID_50_ per well) as described in Methods. After 4 and 7 days, total DNA was extracted and the amount of total HIV-1 DNA (copies/10^6^ cells) was determined by qPCR. The results from 1 representative donor out of 4 tested are shown. **(B)** MDM were treated with different concentrations of anti-CCL2 or control Ab (2.5, 1.25 and 0.625 μg/ml) for 20 h and then infected as in A. Total DNA was extracted 7 days after infection and the amount of total HIV-1 DNA was determined by qPCR. **(C, D, E)** MDM were treated and infected as in A. Total DNA was extracted 7 days after infection and the amounts of total HIV-1 DNA (C), HIV-1 2-LTR circles **(D)** and integrated HIV-1 DNA **(E)** were determined by qPCR. Data represent mean values (+SE) of the results obtained with 3 (B and D), 4 (E) and 13 (C) different donors. *p < 0.05; **p < 0.01; ***p < 0.001.

To further investigate the mechanisms underlying the inhibition of HIV-1 replication associated with CCL2 neutralization in MDM, we quantified the levels of viral DNA that accumulated at different time points post-infection. Toward this aim, MDM exposed for 20 h to anti-CCL2 or control Ab were challenged with HIV-1_BaL_, and total viral DNA was quantified 4 and 7 days post-infection. A marked variability was observed in the levels of total HIV-1 DNA among donors. In particular, in untreated MDM the values of viral DNA copies per 10^6^ cells ranged from 8,301.6 to 155,264.4 [median value: 73,824.5 ± 37,301.1 (SE)] and from 69,140.2 to 258,503.4 [median value: 137,845.2 ± 42,908.1 (SE)] at 4 and 7 days post-infection, respectively (n = 4). Despite this variability, treatment of MDM with anti-CCL2 Ab led to a significant decrease in the accumulation of HIV-1 DNA copies at both time points (Figure 3A), with a more profound reduction of viral DNA levels at 7 days post-infection. These experiments also revealed that CCL2 neutralization strongly influenced the kinetics of HIV-1 DNA accumulation. In fact, as shown in Additional file 4: Figure S4, a marked increase of total viral DNA level was observed in both untreated and control Ab treated cells at 7 *vs.* 4 days post-infection [5.40 ± 2.49 (SE) and 5.60 ± 2.69 (SE) fold, respectively]. In contrast, in anti-CCL2 Ab treated MDM the levels of HIV-1 DNA at 7 days post-infection were similar to those found 4 days after infection [0.96 ± 0.45 (SE) fold].

We next examined the concentration-dependency of the effect of anti-CCL2 Ab on HIV-1 DNA levels after 7 days of infection. As shown in Figure 3B, the viral DNA decrease caused by anti-CCL2 Ab was concentration dependent and statistically significant at all the concentrations tested [0.08 ± 0.03 (SE), 0.12 ± 0.04 (SE) and 0.37 ± 0.07 (SE) at the concentrations of 2.5 (p < 0.001), 1.25 (p < 0.01) and 0.625 (p < 0.05) μg/ml, respectively; n = 3]. The decrease of HIV-1 DNA induced by the maximal Ab concentration was significantly higher with respect to that observed at the lowest one (p < 0.01). In spite of an occasional effect of control Ab on the level of HIV-1 DNA, the presence of anti-CCL2 Ab significantly reduced the amount of viral DNA copies accumulated 7 days post-infection with respect to control Ab treatment [0.26 ± 0.09 (SE) fold *vs.* control Ab; p < 0.001; n = 13] (Figure 3C).

Furthermore, we observed that CCL2 neutralization similarly affected the accumulation of different intracellular forms of HIV-1 DNA. In particular, as shown in Figure 3D, the presence of anti-CCL2 Ab significantly reduced the amount of episomal 2-long terminal repeat (2-LTR) DNA circles accumulated 7 days post-infection with respect to control Ab treatment [0.26 ± 0.14 (SE) fold *vs.* control Ab; p < 0.05; n = 3]. Finally, a marked reduction of integrated proviral DNA, as measured by Alu-PCR, was also observed in anti-CCL2 Ab treated MDM in comparison to control cells [0.28 ± 0.007 (SE) fold *vs.* control Ab; p < 0.001; n = 4; Figure 3E].

Overall, these results clearly indicate that CCL2 neutralization significantly impairs HIV-1 DNA accumulation.

### SAMHD1 expression and function are not affected by CCL2 neutralization in infected MDM

**Figure 4:**
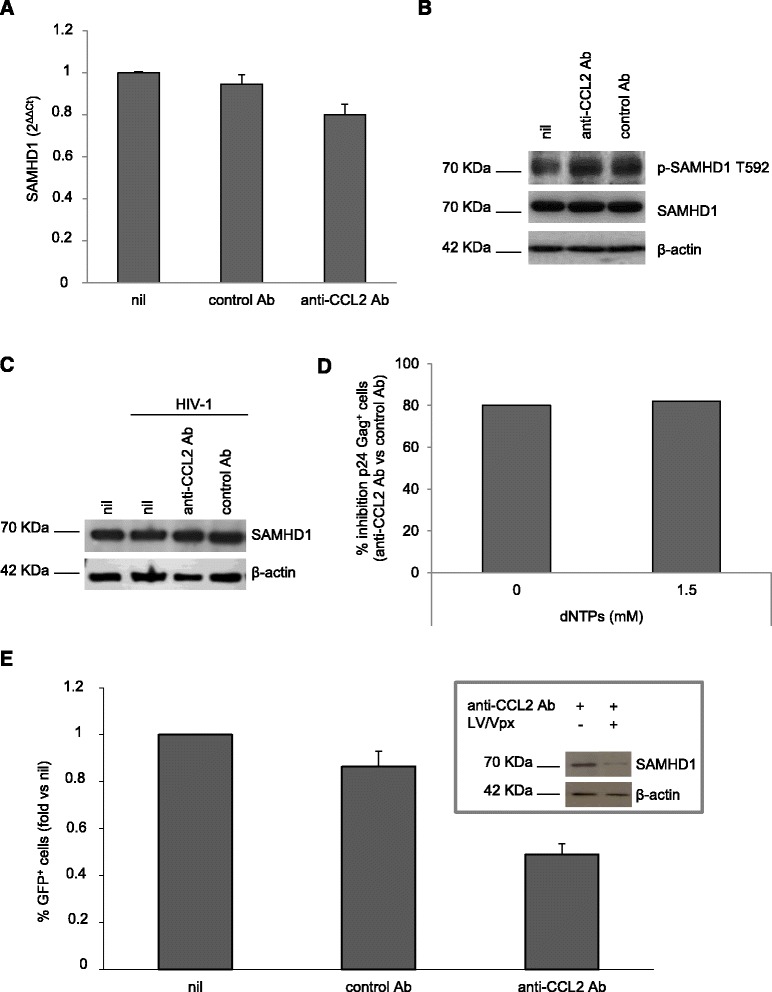
**SAMHD1 expression and function are not affected by CCL2 neutralization in MDM. (A)** MDM were treated with anti-CCL2 or control Ab (2.5 μg/ml) for 20 h. Total RNA was then extracted, retrotranscribed, amplified and the 2^ΔΔCt^ values for SAMHD1 transcripts were calculated as described in Methods. **(B)** MDM were treated as in A and SAMHD1 protein expression and phosphorylation in whole cell extracts were detected by western blot. Actin was used as house-keeping gel loading control. **(C)** MDM were treated as in A and then infected with HIV-1_BaL_ (3000 TCID_50_ per well) as described in Methods. Cells were lysed 14 days after infection and SAMHD1 protein expression in whole cell extracts was detected by western blot. Actin was used as house-keeping control. **(D)** MDM were treated and infected as in C either in the absence or in the presence of dNTPs (1.5 mM) as described in Methods. After 14 days, cells were recovered and HIV-1 Gag expression was evaluated by flow cytometry. **(E)** MDM were treated as in A and then challenged with LV/Vpx expressing GFP (2 × 10^6^ TU per 10^6^ cells). The proportion of GFP^+^ cells was determined 3 days post-infection by flow cytometry. The effect of Vpx on SAMHD1 protein expression is shown in the inset. In A and E, data represent mean values (+SE) of the results obtained with 4 different donors. In B, C and D, the result from a single representative experiment out of 4 independently performed is shown.

Searching for potential correlates of the post-entry restriction of the HIV-1 life cycle mediated by CCL2 blocking in MDM, we firstly focused our attention on SAMHD1, a host factor that restricts HIV-1 replication in myeloid cells by either depleting dNTP levels below those required for optimal synthesis of HIV-1 DNA or degrading viral RNA [33,35]. Therefore, we performed qPCR and Western blot analysis to evaluate SAMHD1 expression in both control and anti-CCL2 Ab treated MDM at the time of HIV-1 infection (i.e., 20 h after treatment). As shown in Figure 4A, treatment of MDM with anti-CCL2 Ab did not significantly affect SAMHD1 gene expression. These results were further confirmed by Western blot analysis. As shown in Figure 4B, SAMHD1 protein was highly expressed in control MDM, and the presence of CCL2 Ab does not change either its expression or the level of its phosphorylation, that has been shown to regulate the RNase activity of this enzyme [35]. Similarly, SAMHD1 protein expression was not affected by CCL2 blocking in HIV-1-infected MDM (Figure 4C). These findings strongly suggest that the mechanism of inhibition of HIV-1 replication induced by anti-CCL2 Ab do not involve modulation of SAMHD1 expression or phosphorylation.

We further investigated whether SAMHD1 dNTPase activity could be regulated by CCL2 blocking. To this aim, we evaluated the effect of dNTPs supply in our experimental conditions. MDM exposed for 20 h to anti-CCL2 or control Ab were infected with HIV-1_BaL_ either in the absence or in the presence of dNTPs, and the percentage of p24 Gag^+^ cells was assessed 14 days post-infection. As expected, exogenous dNTPs increased the percentage of p24 Gag^+^ cells in all conditions (Additional file 5: Figure S5). Interestingly, the percentage of p24 Gag^+^ cells was similarly reduced by anti-CCL2 Ab both in the absence and in the presence of exogenous dNTPs (Figure 4D). These results indicate that SAMHD1 dNTPase activity is not involved in the restriction of HIV-1 replication mediated by CCL2 blocking.

Since it has been shown that SIV-Vpx induces proteasomal degradation of SAMHD1 [30,31], we utilized an HIV-1 based lentiviral vector (LV) containing SIV-Vpx and expressing GFP (LV/Vpx) to knock-down SAMHD1 and then compare LV/Vpx infected MDM treated with anti-CCL2 or control Ab. MDM exposed for 20 h to anti-CCL2 or control Ab were challenged with LV/Vpx, and the proportion of GFP^+^ cells was determined 3 days post-infection by flow cytometry. As shown in Figure 4E, a significant reduction in the fraction of GFP^+^ cells was observed in the cultures treated with anti-CCL2 Ab. As expected, the presence of Vpx strongly reduced SAMHD1 protein expression (Figure 4E, inset), thus confirming that the observed inhibition is SAMHD1 independent. These results indicate that CCL2 neutralization is still capable of inhibiting HIV-1 infection in MDM when SAMHD1 expression is strongly reduced.

Therefore, neither altered SAMHD1 expression nor function likely account for the CCL2 neutralization-dependent restriction of HIV-1 replication in MDM.

### Neutralization of CCL2 selectively up-regulates A3A expression in MDM

**Figure 5:**
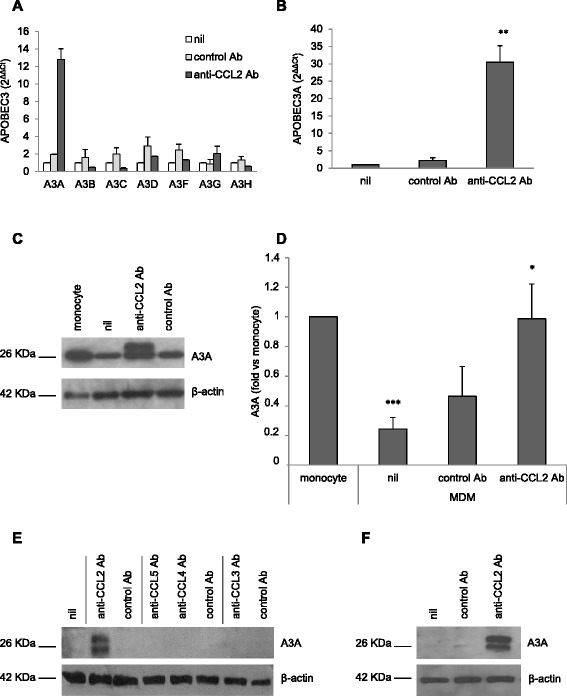
**Endogenous CCL2 neutralization induces A3A expression in MDM. (A-B)** MDM were treated with anti-CCL2 or control Ab (2.5 μg/ml). After 20 h, total RNA was extracted and A3 expression was analyzed by qPCR and expressed as 2^ΔΔCt^ values. In A, the result from 1 representative donor of 3 tested is shown. In B, data represent mean values (+SE) of the results obtained with 6 donors. **p< 0.01 (anti-CCL2 Ab vs. nil or control Ab). **(C-D)** MDM were treated as in A, and A3A protein expression was detected by western blot and compared to those of freshly isolated monocytes from the same donor. In C, the result from 1 representative donor of 5 analyzed is shown. In D, the ratio of A3A to actin was determined by densitometry, and graph shows the mean of the fold relative to monocytes (+SE) of the 5 donors tested. *p< 0.05 (anti-CCL2 Ab vs. nil); ***p< 0.001 (nil vs. monocyte). **(E-F)** MDM were treated as in A in the presence or absence of IFN-α (1:750) or IFN-β (1:250) neutralizing serum, and A3A protein expression was detected by western blot. In E, the result from 1 representative donor of 5 tested is shown. In F, the ratio of A3A to actin was determined by densitometry, and graph shows the mean of the fold relative to nil (+SE) of the 5 donors tested. **(G)** MDM were treated with anti-CCL2, anti-CCL3, anti-CCL4, anti-CCL5 and control Ab (2.5 μg/ml). After 20 h, cells were lysed and A3A protein expression was detected by western blot. The result from 1 representative donor of 6 tested is shown. **(H)** MDM were treated as in A and then infected with HIV-1BaL. After 14 days, A3A protein was detected by western blot. The result from 1 representative donor of 6 tested is shown.

We next focused our attention on the A3 family of restriction factors in that some of these proteins restrict the infectivity of HIV-1 primarily by hyper mutating viral cDNA and inhibiting reverse transcription and integration, thus impairing viral DNA accumulation [50,51]. Therefore, we performed qPCR analysis for A3 family member transcripts in both control and anti-CCL2 Ab treated MDM at the time of HIV-1 infection (i.e., 20 h after treatment). As shown in Figure 5A, treatment of MDM with anti-CCL2 Ab strongly increased the accumulation of A3A transcripts, whereas A3B, A3C, A3D, A3F, A3G and A3H mRNA levels were not significantly affected. We further investigated the consequence of CCL2 neutralization on A3A mRNA expression by using MDM obtained from additional donors. The effect of CCL2 blocking on A3A transcripts was consistently observed in all the donors analyzed. In particular, as shown in Figure 5B, a 30.45 ± 4.70 (SE) fold increase in A3A mRNA level was observed after 20 h of exposure to anti-CCL2 Ab (p < 0.01 *vs.* nil or control Ab; n = 6).

We then studied whether the increase in A3A mRNA levels corresponded to induction of A3A protein expression. As previously reported by other groups [52,53], two isoforms of A3A can be detected by Western blot analysis in MDM (Figure 5C), with some variability in their constitutive expression among donors. In particular, some donors did not constitutively express both A3A isoforms, whereas others expressed the isoform with a lower molecular weight (data not shown). Despite this variability, anti-CCL2, but not control Ab, resulted in a strong induction of both A3A isoforms expression as measured after 20 h of incubation (Figure 5C). Interestingly, A3A expression levels induced by CCL2 blocking were comparable to those of freshly isolated monocytes (Figure 5C and D).

Furthermore, to exclude any effect of CCL2 neutralization on A3G expression, we analyzed A3G protein levels by Western blot analysis. As shown in Additional file 6: Figure S6, MDM did not express the low molecular mass form of A3G (~46 kDa) either in control conditions or after incubation with anti-CCL2 Ab, whereas this protein was induced by IFN-β, as already reported by others [54].

It has been previously reported that IFNs are potent inducers of A3 family member expression [43]. In particular, type I IFN has been shown to induce A3A expression in monocytes/macrophages [42,52,53]. We thus investigated whether the induction of A3A expression elicited by CCL2 neutralization was mediated by type I IFN. To this aim, endogenous IFN, eventually produced in MDM exposed to anti-CCL2 Ab, was neutralized by means of Ab to IFN-α or IFN-β. As shown in Figure 5E and F, the presence of these Abs did not modify A3A protein expression induced by CCL2 blocking. In keeping with this result, type I IFN was not detected in the supernatants of anti-CCL2 Ab-treated MDM as measured by a cytopathic effect reduction assay (data not shown). These results indicate that A3A induction by CCL2 neutralization is type I IFN independent.

Moreover, we investigated whether A3A expression was specifically induced by CCL2 blocking. To this aim, we targeted other CC-chemokines which are expressed by macrophages and are further induced by HIV-1 infection of these cells [17]. As shown in Figure 5G, treatment of MDM with anti-CCL3, anti-CCL4 and anti-CCL5, as well as control Ab, did not affect A3A protein expression.

Finally, we investigated whether A3A induction by CCL2 neutralization also occurred in HIV-1 infected MDM. Therefore, MDM exposed for 20 h to anti-CCL2 or control Ab were infected with HIV-1_BaL_ and A3A expression was analyzed 14 days post-infection by Western blot analysis. As shown in Figure 5H, A3A was not detected in MDM infected with HIV-1 either in the absence or in the presence of control Ab, whereas this protein was highly expressed in infected cells maintained in the presence of anti-CCL2 Ab.

Since controversial data have been reported by different groups concerning the effect of Vpx on A3A expression [55-57], we analyzed the expression of this protein in MDM challenged with LV/Vpx. As shown in Additional file 7: Figure S7, unlike SAMHD1 expression, the presence of Vpx in lentiviral preparations did not affect A3A expression.

### Effect of the timing of anti-CCL2 Ab addition on HIV-1 replication restriction and A3A expression

**Figure 6:**
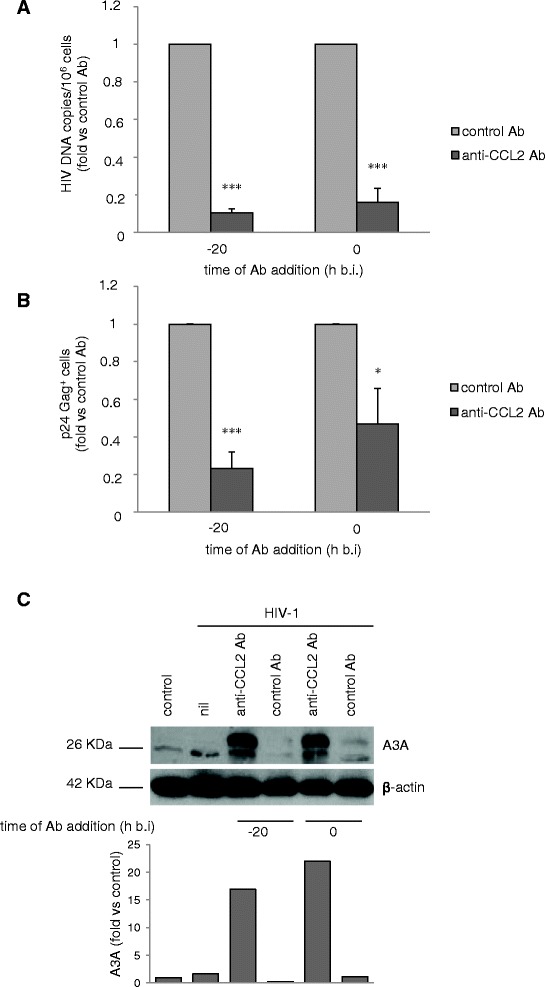
**Effect of the timing of anti-CCL2 Ab addition on HIV-1 replication and A3A expression.** MDM were treated with anti-CCL2 or control Ab (2.5 μg/ml) 20 h before or at the time of infection with HIV-1_BaL_ (3000 TCID_50_ per well) as described in Methods. **(A)** Total DNA was extracted 7 days after infection and the amount of total HIV-1 DNA (copies/10^6^ cells) was determined by qPCR. Data represent mean values (+SE) of the results obtained with 3 different donors. **(B)** Cells were recovered 14 days after infection and HIV-1 Gag expression was evaluated by flow cytometry. Data represent mean values (+SE) of the results obtained with 7 different donors. *p < 0.05; ***p < 0.001. **(C)** Cells were lysed 14 days after infection, and A3A protein expression in whole cell extracts was detected by western blot. Actin was used as house-keeping control. The ratio of A3A to actin protein was determined by densitometry, and graph shows the fold relative to control. The result from one representative experiment of 4 independently performed is shown.

We then investigated whether the timing of anti-CCL2 Ab addition to MDM was important for the impairment of HIV-1 replication. Therefore, MDM were treated with anti-CCL2 Ab 20 h before or at the time of infection, and viral DNA accumulation and the proportion of p24 Gag^+^ cells were measured 7 and 14 days post-infection, respectively. As shown in Figure 6A, a decrease of HIV-1 DNA accumulation was observed in MDM exposed to anti-CCL2 Ab both 20 h before or at the time of infection [0.10 ± 0.02 (SE) and 0.16 ± 0.07 (SE) fold *vs.* control Ab, respectively (p < 0.001); n = 3]. Furthermore, addition of anti-CCL2 Ab concomitantly with HIV-1 infection also significantly reduced the percentage of p24 Gag^+^ cells, although to a lesser extent with respect to what observed in MDM exposed to anti-CCL2 Ab 20 h before infection [0.23 ± 0.08 (SE) and 0.47 ± 0.19 (SE) fold *vs* control Ab in MDM treated 20 h before (p < 0.001) or at the time of infection (p < 0.05), respectively; n = 7] (Figure 6B).

Finally, we determined whether A3A expression was also induced in infected MDM exposed to anti-CCL2 Ab at the time of infection. Notably, as shown in Figure 6C, A3A protein was induced in these experimental conditions at similar levels to those observed in cells exposed to anti-CCL2 Ab 20 h before infection.

Therefore, these results further highlights the association between A3A expression and the restriction of HIV-1 replication induced by neutralization of endogenously released CCL2.

### Transcriptome analysis of the effect of CCL2 neutralization on global gene expression in MDM

To characterize the effect of CCL2 blocking on global gene expression, MDM from two different donors were exposed to anti-CCL2 Ab, and compared to control samples. Cells were collected at 20 h post-treatment and total RNA was isolated, subjected to poly(A) selection followed by reverse transcription, generation of cDNA libraries, and sequencing.

We performed differential expression analysis using DESeq2 [58] and identified 194 genes upregulated upon treatment and 117 downregulated (FDR < 0.01 and fold change > 2) (Additional file 8: Table S1).

**Figure 7:**
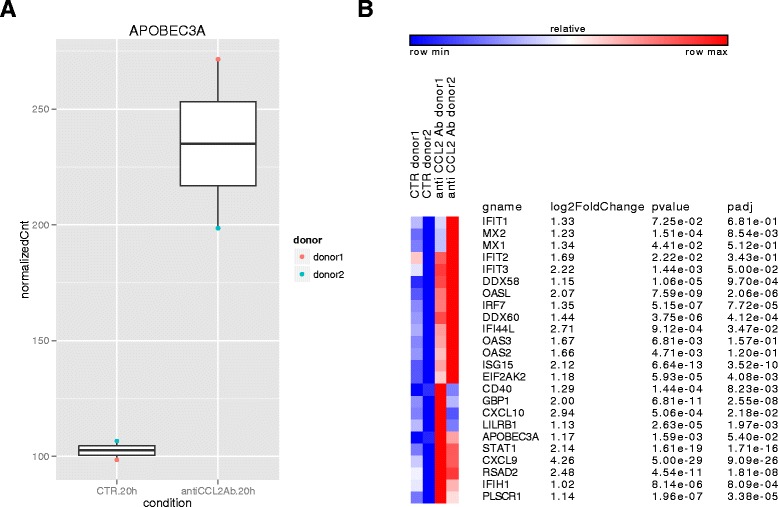
**Transcriptome analysis of the effect of CCL2 neutralization.** MDM were treated or not with anti-CCL2 Ab (2.5 μg/ml) for 20 h. Total RNA was then extracted, subjected to poly(A) selection followed by reverse transcription, generation of cDNA libraries, and sequencing. **(A)** Dotplot of DESeq2 normalized counts of the *A3A* gene following 20 h exposure to anti-CCL2 Ab. Each condition has two biological replicates collected from two donors. Dots were colored by donors. **(B)** Heatmap of 24 defense-response-to-virus genes which are upregulated two-fold in 20 h anti-CCL2 Ab versus the 20 h control MDM. Heatmap were made by GENE-E (http://www.broadinstitute.org/cancer/software/GENE-E).

In keeping with the data shown in Figure 5, we found that the *A3A* gene was more highly expressed in the 2 treated donors analyzed, compared to their controls (Figure 7A). In order to gain further insight into the specific categories of genes whose expression is changed upon treatment, we performed annotation and pathway analysis. According to Ensembl75 annotation, 111 genes were detected in our RNAseq data to be annotated with the GO biological term “defense response to virus” (GO:0051607) and 24 out of these were more than 2 fold up in MDM treated with anti CCL2 Ab for 20 h with respect to control cells (Figure 7B). More GO annotation was done to our 194 upregulated genes using DAVID v6.7 [59,60] and these genes were significantly enriched in GO biological process terms GO:0009615 ~ response to virus (p value = 6.19E-09) and GO:0004587 ~ innate immune response (p value = 7.55E-07) (Additional file 9: Table S2).

**Figure 8:**
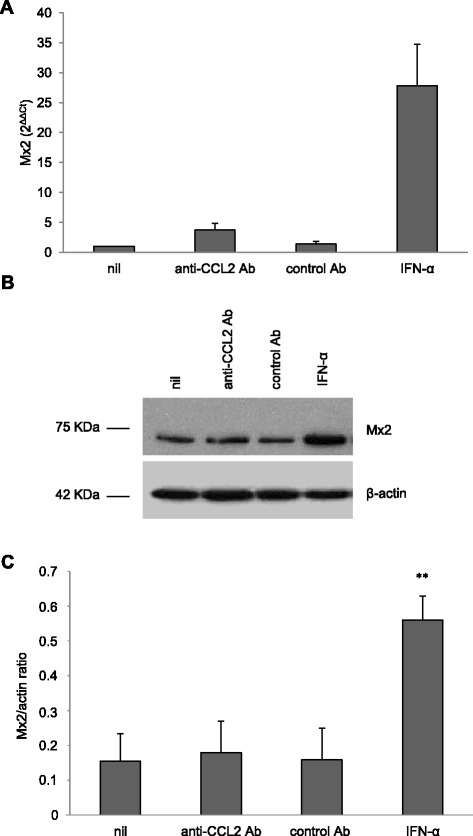
**Effect of CCL2 neutralization on Mx2 expression.** MDM were treated with anti-CCL2 or control Ab (2.5 μg/ml) or IFN-α (1000 U/ml) for 20 h. **(A)** Total RNA was extracted, retrotranscribed, amplified and the 2^ΔΔCt^ values for Mx2 transcripts were calculated as described in Materials and Methods. The result from a single representative experiment of 3 independently performed is shown. **(B)** Cells were lysed and Mx2 protein expression in whole cell extracts was detected by western blot. The result from a representative donor out of 6 analyzed is shown. **(C)** Densitometric analyses of Mx2 expression performed on immunoblotting of MDM extracts from the 6 different donors analyzed. The graph shows the ratio of Mx2 to actin OD determined by densitometry. **p < 0.01 (IFN-α *vs.* nil).

Besides A3A, the *Mx2* gene was among the genes up modulated by CCL2 in Figure 7B. Mx2 is a type I IFN-induced protein that has been recently shown to restrict HIV-1 replication by inhibiting the nuclear accumulation and integration of HIV-1 reverse transcripts [61]. We thus analyzed the effect of CCL2 neutralization on the expression of *Mx2* gene by RT-qPCR in MDM obtained from additional donors. As shown in Figure 8A, exposure of MDM to anti-CCL2 Ab induced an increase in Mx2 transcript accumulation that was considerably lower than that elicited by IFN-α. We thus analyzed Mx2 protein levels by Western blot analysis. As shown in Figure 8B and C, the expression of this protein was not modified by CCL2 neutralization, whereas it was induced by IFN-α.

## Discussion

CCL2 is a potent pro-inflammatory chemokine induced during HIV-1 infection and believed to be one of the key factors driving chronic inflammation and tissue damage in infected individuals [18,19]. Studies conducted thus far have shown a multifaceted impact of CCL2 in HIV-1 pathogenesis and disease progression. In particular, CCL2, mainly produced by monocytes/macrophages both *in vitro* and *in vivo*, recruits CCR2^+^CD4^+^ T lymphocytes and monocytes/macrophages, which represent key target cells for HIV-1 infection. This chemokine also enhances viral replication in T cells by increasing CXCR4 expression and HIV-1 entry [62]. *In vivo*, high CCL2 levels in plasma and cerebrospinal fluid of HIV-infected individuals correlate with viral load [63-66]. Moreover, a genetic polymorphism in the *CCL2* gene (2518G/A), linked to an increased production of the protein, is associated with increased risk of HIV-1 infection, disease progression and development of HIV-associated dementia [67]. Finally, the increase in plasma viremia observed in acutely HIV-infected individuals is associated with a rapid and sustained elevation in CCL2 plasma levels [68].

We have previously found that CCL2 expression was up-regulated during monocyte differentiation to macrophages and it was further increased by HIV-1 infection; conversely, endogenously released CCL2 promoted HIV-1 replication [21,69]. Consistently, infection of MDM with HIV-1 in the presence of anti-CCL2 Ab resulted in a potent inhibition of p24 Gag Ag release with respect to control cells, in the intracellular accumulation of this viral antigen and in remarkable changes in cell morphology and size [21]. Thus, CCL2 may represent an autocrine factor important for enhancing virion production likely by affecting the macrophage cytoskeleton. The present study indicates that additional, early post-entry steps of the HIV-1 life cycle are also impaired upon CCL2 blocking. In fact, we report here that neutralization of CCL2 potently reduces the proportion of p24 Gag^+^ cells during the course of either a productive infection with R5 HIV-1_BaL_ or a single cycle infection with (VSV-G) HIV-1. Since neither HIV-1 based VLP entrance nor reverse transcription immediate products levels are affected by CCL2 blocking, the mechanism of CCL2 neutralization-mediated restriction occurs independently of HIV-1 entry and RT activity. Conversely, CCL2 blocking potently limits the accumulation of viral DNA, both total, integrated and 2-LTR circles.

In the present study we also provide evidence that endogenous CCL2 may represent an autocrine factor acting as a negative regulator of A3A expression in macrophages. In fact, targeting CCL2 induces A3A mRNA and protein expression to levels observed in freshly isolated monocytes. This effect is specific for A3A among the A3 family of cytidine deaminases, since the expression of the other A3 enzymes, including the most characterized anti-HIV protein A3G, is not induced by CCL2 blocking. Interestingly, the increase of A3A expression elicited by CCL2 neutralization in MDM occurs independently of type I IFN. The identification of an IFN independent pathway of A3A induction is of significance, particularly in the context of viral infection. Most viruses, including HIV-1, are known to evade the host immune response by blocking IFN-mediated anti-viral responses [70]. Intriguingly, it has been shown that SIV infected astrocytes produce CCL2 that binds to the CCR2 receptors on macrophages resulting in suppression of specific IFN stimulated genes [71]. Our finding, showing that the A3A antiviral response can be activated through a mechanism other than the IFN-driven one, unravels a novel pathway which may contribute to regulate the expression of this enzyme *in vivo*.

In addition to CCL2, other CC chemokines, namely CCL3, CCL4 and CCL5, are constitutively released by MDM and their production can be further increased following HIV-1 infection of these cells [17]. Our data indicate that neutralization of these chemokines does not affect A3A protein levels. Thus, modulation of A3A expression seems to be a CCL2 specific task and not a general function of chemokines. CCR2 is the only CCL2 receptor described thus far. Two different isoforms of this receptor, named a and b, have been identified. They represent alternatively spliced variants of a single gene that differ only in their carboxyl tails. In a previous study we demonstrated that monocyte differentiation to macrophages resulted in a decrease of CCR2 mRNA, particularly the b isoform, as well as of CCR2 levels at the plasma membrane. The decrease of CCR2 expression was associated with a reduced biological response of MDM to CCL2 in terms of calcium flux and migration [69]. In the present study, the role of CCR2 in the CCL2-mediated modulation of A3A expression was not investigated. However, we speculate that low levels of CCR2 expression could mediate the observed phenotype. Furthermore, both increased CCR2 expression and enhanced response to CCL2 have been described in HIV-1-infected leukocytes by other groups [72,73]. Thus, it cannot be excluded that enhanced CCR2 expression in HIV-1 infected macrophages could contribute to the observed effects on viral replication and A3A expression. To our knowledge, this is the first study showing that an endogenous chemokine can act as a negative regulator of A3 protein expression. In this regard, previous studies reported that triggering of CCR5 or CCR6 by CCL3 and HSP70 or CCL20 and human β-defensin 2, respectively, supported A3G expression. Interestingly, in these studies the increase in A3G levels was associated with inhibition of HIV-1 replication in CD4^+^ T lymphocytes and dendritic cells (DC) [74,75].

Since the identification of A3 cytidine deaminases as HIV-1 antiviral factors, a considerable amount of studies have explored the role of these proteins in the pathogenesis of HIV-1 infection and the underlying mechanisms of action. Cells belonging to the myeloid lineage express all members of the A3 family, but A3A is the only one restricted to these cells among the white blood cells targets of HIV-1 [40,41]. A3A shares sequence and functional homology with A3G and is a potent inhibitor of retrotrasposons, Parvovirus and HTLV-1 [76,77]. Its role as inhibitor of HIV-1 replication is controversial. In fact, although past studies indicated that A3A does not exert an anti-HIV activity in established cell lines in which it is ectopically expressed [36], more recent studies revealed that an anti-HIV activity is explicit only in cells in which A3A is naturally expressed, i.e. myeloid cells. In this regard, Berger and colleagues reported that the pool of A3A present in primary macrophages, DCs and differentiated THP-1 cells (that mimic macrophages upon differentiation) is directly capable of inhibiting incoming viruses at the reverse transcription step. In that study, A3A expression has been shown to be up-regulated in MDM exposed to HIV-1 [56]. We do not observe such effect in our infected MDM cultures, in which A3A is increased over the basal level only as a consequence of CCL2 neutralization. The apparent discrepancy between these results and ours can be, at least in part, explained by the different protocol used for monocyte culture, which may greatly influence the ability of MDM to activate innate immune reactions and support HIV-1 replication. To this regard, it has been recently shown that MDM generated in the presence of M-CSF are more permissive to HIV-1 replication, but paradoxically have a strongest innate immune response [78]. Furthermore, additional studies pointed to an up-regulated expression of A3A as the causal factor of the restriction of HIV-1 replication elicited by different conditions in myeloid cells [53,79-81]. In particular, exposure to exogenous type I IFNs, or to the type I IFN-inducing cytokine IL-27, has been shown to stimulate A3A expression in both macrophages and DCs and to inhibit HIV-1 replication in the former and viral spread to CD4^+^ T cells in the latter [79,80]. In macrophages, treatment with IFN-α resulted in a strong induction of A3A expression and reduction of HIV-1 DNA accumulation, associated with an increased G-to-A editing of viral DNA [53]. Finally, Cassetta and co-workers reported that restriction of HIV-1 replication in M1 polarized macrophages was associated with the up-regulated expression of A3A, but not A3G [81].

Although in this study we demonstrate that CCL2 neutralization impairs HIV-1 DNA accumulation, the cellular factors that sustain this inhibition remain to be identified and the mechanisms deciphered. Keeping in mind the effect on viral DNA accumulation, a role for host restriction factor(s) affecting this step of the HIV-1 life cycle might be suggested. In this regard, we excluded the involvement of SAMHD1 in this phenomenon. In fact, our data strongly indicate that neither altered SAMHD1 expression nor function likely account for the restriction of HIV-1 replication mediated by CCL2 neutralization. Although SAMHD1 has been shown to reduce the pool of dNTPs thus inhibiting the reverse transcription step especially in myeloid cells, a recent study reported that this factor moderately restricts a macrophage-tropic HIV-1 strain in MDM, whereas it potently inhibits HIV-1 replication in undifferentiated monocytes [82]. In accordance with this report, it has been shown by other groups that SAMHD1 is not a major effector of the early type I IFN-mediated block toward HIV-1 [57,83]. Furthermore, our data indicate that CCL2 blocking does not affect the expression of Mx2, recently shown to restrict HIV-1 replication by inhibiting the nuclear accumulation and integration of HIV-1 reverse transcripts [61]. Conversely, we found a strong association between CCL2 neutralization-mediated inhibition of HIV-1 replication and induction of A3A expression. Interestingly, the levels of A3A expression elicited by CCL2 blocking in MDM are comparable to those of freshly isolated monocytes, where this enzyme has been demonstrated to restrict HIV-1 infection [42]. In keeping with these data, other groups reported that inhibition of HIV-1 DNA accumulation in myeloid cells was correlated to A3A expression in different conditions [53,81]. However, since our efforts to deplete A3A protein in anti-CCL2 Ab-treated MDM using siRNA have not been successful, as already experienced by other groups in different experimental settings [53,84], we could not formally prove the involvement of this protein in the observed inhibition of viral replication. Thus far, we cannot rule out the possibility that other not yet identified factors may be involved in the restriction of HIV-1 replication elicited by CCL2 blocking. In this regard, our data show that other genes related to the defense response to viruses are upmodulated in MDM exposed to anti-CCL2 Ab, thus suggesting that several factors might contribute to the restriction of HIV-1 replication elicited by CCL2 neutralization. Future work is required to define whether the CCL2 blocking-mediated inhibition of HIV-1 replication in MDM is associated or not with an increased G-to-A editing of viral DNA and/or with others mechanisms of HIV-1 restriction.

## Conclusions

Neutralization of macrophage-derived CCL2 potently restricts HIV-1 replication by inhibiting early post-entry steps of the viral life cycle. Since macrophages represent potential viral reservoirs capable of producing replication-competent HIV-1 virions even in the presence of effective combination antiretroviral therapy, targeting CCL2 may represent a novel therapeutic strategy for enhancing cellular defences against HIV-1 and protecting macrophages from infection. Interestingly, enhanced CCL2 expression has been also found in ectocervical tissue explants following HIV-1 infection, and neutralization of the chemokine in this experimental system has been shown to result in viral transcription decrease [85]. Thus, since the heterosexual route through female reproductive tract mucosal surfaces is the predominant mode of HIV-1 transmission, targeting CCL2 may offer a tantalizing possibility of leveraging innate immunity to fend off viral infection at the entry route.

## Methods

### Ethics statements

Healthy donor Buffy coats were obtained from Centro Trasfusionale-University of Rome “Sapienza”. Buffy coats were not obtained specifically for this study. Informed consent has not been asked because data were analyzed anonymously. Data from healthy donors have been treated by Centro Trasfusionale according to the Italian law on personal data management “Codice in materia di protezione dei dati personali” (Testo unico D.L. June 30, 2003 n. 196).

### Cell separation and culture

Monocytes were isolated from peripheral blood mononuclear cells obtained from healthy donor Buffy-coats by positive immunomagnetic selection using CD14 micro beads (MACS monocyte isolation kit, Miltenyi Biotec), according to the manufacturer’s instructions. This procedure yields a 95-98% pure population of monocytes, as assessed by fluorescence-activated cell sorter analysis of lineage-specific surface markers (CD1a, CD14, CD3, CD19, and CD56). Freshly isolated monocytes were seeded in 48-well cluster plates (1x10^6^ cells per well in 1 ml) and cultured for 6 days in endotoxin-free IMDM (Life Technologies) containing 10% FBS to obtain MDM as previously described [69].

### Reagents

Rabbit polyclonal and mouse monoclonal Abs directed against CCL2 and CCL3, respectively, as well as control rabbit and mouse Abs, were purchased from PeproTech. Goat anti-CCL4, anti-CCL5 and control Abs were purchased from R&D Systems. At the concentration employed in the study, the anti-CCL3 and anti-CCL5 Abs effectively neutralized the endogenously produced chemokines, whereas the anti-CCL4 Ab inhibited CCL4-mediated ERK1/2 phosphorylation, as assessed by Elisa and western blot, respectively (data not shown). Recombinant human IFN-β and IFN-α were kindly provided by Serono and Schering-Plough, respectively. Sheep anti-human IFN-α (neutralizing titer is 1:3000000 against 8 units IFN-α) and calf anti-human IFN-β (neutralizing titer is 1:25000 against 10 units IFN-β) sera were kindly provided by Dr Vilcek. In a cytopathic effect reduction assay these sera have been shown to neutralize exogenous IFNs at the used concentrations (data not shown). Soluble LDLR was purchased from R&D Systems. The nucleoside analogue reverse-transcriptase inhibitor AZT and the fusion inhibitor T20 were obtained from the National Institutes of Health AIDS Research and Reference Reagent Program.

### Viruses and infection

HIV-1 preparations pseudotyped with VSV-G were obtained from the supernatants of 293 T cells 48 h after cotransfection with a vector expressing VSV-G under the control of CMV immediate-early promoter and a vector expressing pdeltaEnvNL4-3 HIV-1 in a molar ratio of 1:5. Cotransfection was performed using Lipofectamine 2000 (Invitrogen). Supernatants were clarified and concentrated by ultracentrifugation as previously described [86]. For lentiviral preparations a total of 12 μg of plasmid DNA were used for each plate in a ratio 6:4:2 (transfer vector: packaging vector: VSV-G vector). The plasmids pCMVdR8.2, the VSV-G envelope expressing pMD.G plasmid and the pTY2-CMV-GFP-W transfer vector have been already described [87-89]. For construction of SIV-Vpx expressing plasmid, Vpx derived from the SIVMAC251 strain was modified [90] and 10 μg of this plasmid were included in the transfection. 293 T cells were transiently transfected with the calcium phosphate-based protocol using the Calcium Phosphate-based Profection Mammalian Transfection System (Promega Corporation) and the supernatants were clarified and concentrated by ultracentrifugation as previously described [88]. VSV-G pseudotyped HIV-1 preparations were titrated in terms of HIV-1 CAp24 content using quantitative enzyme-linked immunosorbent assay (ELISA; Innogenetics), whereas lentiviral titres were normalized by RT [91]. Infection with pseudotyped HIV-1 derivatives was achieved by spinoculation at 400 × *g* for 30 min at room temperature using 250 ng CAp24 equivalent of (VSV-G) HIV-1 per 10^5^ cells or 2×10^6^ TU of LV/Vpx per 10^6^ cells, respectively. Virus adsorption was prolonged for an additional 3 h at 37°C and the cells were washed and re-fed with complete medium. Cells were analyzed 3 days post-infection.

For VLP preparations, 293/RGP cells [92] were co-transfected by liposomes (Lipofectamine 2000) with the immediate-early CMV promoter vector expressing NefG3C-GFP alone or in combination with vectors expressing VSV-G or R5 Env HIV-1 from the Ada isolate. After 6 h, cells were induced with 5 mM sodium butyrate (Sigma-Aldrich) and 2 μM of Ponasterone A (PonA) (Sigma-Aldrich) for 24 h. Supernatants were then replaced with fresh medium containing the inducers. VLP-containing supernatants were harvested from 48 to 72 h later, clarified, and concentrated by ultracentrifugation on 20% sucrose cushion at 100,000 *g* for 2 h at 4°C. VLP preparations were titrated by measuring the HIV-1 CAp24 contents by quantitative ELISA (Abbott, Abbott Park). For MDM challenge, VLPs (1 μg of CAp24 equivalent per 10^5^ cells) were absorbed by spinoculation at 150 *g* for 30 min at room temperature or at 4°C. Afterwards, the cell cultures were re-fed by adding fresh medium, and incubated at 37 or 4°C. After 2 or 4 h, the cells were treated for 15 min with trypsin at 37 or 4°C, fixed, and analyzed by flow cytometry. Representative images were taken by the EVOS FL Cell Imaging System (Life Technologies) and by the Leica TCS SP2 AOBS apparatus (Leica Microsystemsy) for the confocal laser-scanner microscopy analysis.

For productive infection, cells were infected with the Vif^+^ CCR5-dependent HIV-1_BaL_ strain (3000 TCID_50_ per well; Advanced Biotechnologies). After 2 h, cells were washed and maintained in complete medium either in the presence or in the absence of anti-CCL2 or control Ab. Medium was replaced with fresh medium every 7 days. Abs were added twice a week. In some experiments, infected MDM were treated with dNTPs (Pharmacia) once a week.

### ELISA

The levels of CCL2 present in culture supernatants were measured by ELISA kits purchased from R&D Systems (detection limit 5 pg/ml). Viral production was monitored by measuring HIV-1 p24 Gag Ag release by the INNOTEST HIV Antigen mAb kit (Innogenetics).

### Flow cytometry analysis of p24 Gag Ag

MDM were detached by using 0.05% trypsin-EDTA solution, and intracellular HIV-1 p24 Gag expression was evaluated by flow cytometry. Briefly, cells were fixed and permeabilized by using Fixation and Permeabilitazion Buffers (eBioscience). Cells were then labelled with a Phycoerythrin-conjugated monoclonal Ab specific for p24 Gag (KC57-R01, Coulter Clone) and washed with Permeabilization Buffer. The background fluorescence was determined by labelling uninfected cells. Samples were acquired with a FACS Calibur flow cytometer by using Cell Quest (Becton Dickinson) and data analyzed by FlowJo (Tree Star, Inc.) or the Cell Quest software.

### Analysis of HIV-1 DNA synthesis during early phase of transcription by polymerase chain reaction

A semi-quantitative PCR assay was used to estimate the extent of reverse transcription at three replicative steps: R-U5, initial minus strand synthesis; R-PB, initial plus-strand synthesis up to the PB region; and R-gag, complete minus-strand synthesis. The primers used to amplify these products were M667/AA55 (R-U5) producing a 140 bp amplicon, M667/BB301(R-PB) producing a 156 bp amplicon, and M667/M661 (R-gag) producing a 200 bp amplicon (M667, 5′-*GGCTAACTAGGGAACCCACTG*-3′; AA55, 5′-*CTGCTAGAGATTTTCCACACTGAC*-3′; BB301, 5′-*CCCTGTTCGGGCGCCACTG*-3′; M661, 5′-*CCTGCGTCGAGAGAGCTCCTCTGG*-3′). DNA load for each PCR amplification was the same and it was normalized by Real Time PCR quantitative amplification of RNase P gene using TaqMan RNase P Detection Reagents Kit (Applied Biosystems) according to manufacturer’s instructions. Quantization of HIV-1 DNA during PCR amplification was performed by analyzing a standard curve of serial dilutions of HIV plasmid pHxb2 ranging from 1 to 1x10^5^ copies. PCR conditions were: 95°C, 3 min; 95°C, 30 s; 60°C, 30 s; 72°C, 30 s, for 35 cycles.

The quantitative analysis of HIV-1 intermediate reverse-transcript products was performed by SYBR Green real-time PCR of minus-strand strong-stop DNA using M667/AA55 primers and of 1st template switching of RT using M667/BB301 primers. A standard curve was generated using serial dilution ranging from 10,000 to 1 copy of pHxb2 plasmid containing the full length sequence of HIV. Each primer was used at a final concentration of 200 nM. The PCR parameters were as follows: 20 sec at 95°C followed by 40 cycles at 95°C for 15 sec and 60°C for 1 min. Fluorescent product was detected at the last step of each cycle. After amplification a melting curve was generated. All samples and HIV-1 negative controls were run in duplicate.

### Quantification of HIV-1 DNA by real-time polymerase chain reaction

Total DNA was extracted from frozen MDM samples using the QIAamp DNA Blood Mini kit (Qiagen) according to the manufacturer’s instructions; in each extraction uninfected cells were included as negative control. The concentration of the extracted DNA was determined by Real Time PCR quantitative amplification of RNase P gene using TaqMan RNase P Detection Reagents Kit (Applied Biosystems) according to manufacturer’s instructions. Total HIV-1 DNA amount was determined using primers and probe that recognize the HIV-1 *gag* gene [93]. Standard curve was generated using the genomic DNA from the 8E5 cell line, a T lymphoblastoid cell line that contains a single defective copy of HIV-1 genome per cell.

Integrated HIV DNA was quantified by a two-step *Alu-gag* PCR assay. The first PCR was performed in triplicate for each sample, using two primers annealing to Alu sequences (AluFw 5′-*GCCTCCCAAAGTGCTGGGATTACAG*-3′; AluS 5′-*TCCCAGCTACTGGGGAGGCTGAGG*-3′; final concentration: 100nM each) and one primer annealing to *gag* gene (HIV-gag Rev nt 1505–1486: 5′- *GTTCCTGCTATGTCACTTCC*-3′; final concentration: 600nM). Samples were amplified in triplicate also with the HIV-gag Rev primer alone (*gag-only* PCRs) in order to establish whether the level of integration is detectable (see below). Standard curve was generated using serial dilutions of genomic DNA extracted from a standard cell line prepared as previously described [94]. The number of HIV proviruses per cell of the standard cell line was calculated with the same procedure used to calculate total HIV-1 DNA and then adjusting the result by the number of assayed cells calculated by RnaseP gene quantification. The standard curve ranged from 10,000 to 1 integrated HIV-1 copies per well. To keep the number of *Alu* DNA sites constant in each reaction, uninfected PBMC genomic DNA was added to each sample (including standards and controls) to reach the concentration of 70 ng of genomic DNA per well. The parameters for the first PCR were as follows: 10 min at 95°C followed by 25 cycles at 95°C for 15 sec, 50°C for 15 sec and 72°C for 3 min and 30 sec and finally 5 min at 72°C. The second real-time PCR step detects HIV-specific products by using primers annealing to the R and U5 regions within the HIV LTR: RU5 R forward (5′-*TTAAGCCTCAATAAAGCTTGCC*-3′), RU5 U5 reverse (5′-*GTTCGGGCGCCACTGCTAGA*-3′) and RU5 Probe (5′- *CCAGAGTCACACAACAGACGGGCACA*-3′); this real-time PCR was performed on 5 ul from each replicate of both *Alu-gag* and *gag-only* PCRs of standard curve and unknown samples. The PCR parameters were as follows: 3 min at 95°C followed by 45 cycles at 95°C for 3 sec and 60°C for 30 sec. Student’s t-test was performed to determine whether Cts derived from the *Alu-gag* PCRs were significantly lower than those from the *gag-only* reactions. If *Alu-gag* Cts are statistically lower than *gag-only* Cts (p < 0.05) for the same sample, HIV-1 integrated copies can be calculated with reference to the standard curve. Otherwise, the level of integration is to be considered below the limit of detection of the assay [94].

The 2-LTR DNA circles were quantified by SYBR Green real-time PCR and amplified from extracted DNA with 25 nM forward primers HIV-F (*5′-TGTGCCCGTCTGTTGTGTGACT-3′*) and 25 nM reverse primer HIV-R1 (*5′-TGGTGCTACAAGCTAGTACCAGT-3′*) spanning the LTR–LTR junction (modified protocol from Reigadas S et al.) [95]. A plasmid containing the 2-LTR junction sequence was used to generate the standard curve (ranging from 10,000 to 1 copy) for 2-LTR DNA circles quantification; each curve point was obtained diluting the plasmid DNA in 50 ng of genomic DNA from HIV negative donors PBMCs. The PCR parameters were as follows: 20 sec at 95°C followed by 45 cycles at 95°C for 3 sec and 62°C for 1 min. Fluorescent product was detected at the last step of each cycle. After amplification a melting curve was generated. All samples and HIV-1 negative controls were run in triplicate. Rnase-P, HIV-1 *gag* and HIV-1 2LTR standard curves had slopes between −3.15 and −3.6 and the coefficients of correlation was >0.987. HIV-1 DNA load and HIV-1 2-LTR circles load were normalized to the amount of cellular DNA by quantification of RNAseP copies and were expressed as number of copies/10^6^ cells. The limit of detection was 2 copies/10^6^ cells for total HIV-1 DNA and 200 copies/10^6^ cells for HIV-1 2-LTR circles. The ABI Prism 7500 FAST Real time PCR System (Applied Biosystem) was used for PCR amplification, acquisition and data analysis.

### RNA isolation and polymerase chain-reaction

Total RNA was isolated with the RNeasy Mini kit (Qiagen) following the manufacturer’s instructions. RNA was retrotranscribed into cDNA by using poly d(N)_6_ (GE Healthcare) and real-time PCR was performed as previously reported [96]. Validated PCR primers and TaqMan MGB probe (6FAM-labeled) for A3A (Hs00377444), A3B (Hs.PT.53a.20328233), A3C (Hs.PT.53a.4514833), A3D (Hs.PT.53a.4652525), A3F (Hs.PT.53a.27075498), A3G (Hs.PT.49a20797403), A3H (Hs.PT.53a.3752998), SAMHD1 (Hs.PT.49a.21502281) and Mx2 (Hs.PT.58.21491026) were used (A3A from Applied Biosystem, A3B-H, SAMHD1 and Mx2 from Integrated DNA Technologies). As endogenous control, primers and TaqMan probe for the human β-actin (ACTB RNA; Hs99999903_m1; Applied Biosystems) were used. Thermal cycler conditions were previously reported [96]. Relative quantification was performed by using the comparative Ct method as previously described [96].

### RNA sequencing and differential expression analysis

RNAs were collected individually from 2 donors (two 20 h CTR and two 20 h anti-CCL2 Ab). RNAseq libraries were created with the Illumina Truseq RNA sample pre kit and sequenced using the Illumina Hiseq 2500 platform. These 4 samples were sequenced 3 times in multiplexed lanes. Reads of the same sample from 3 runs were pooled together.

Tophat [97] (Version 2.0.6) together with bowtie (version 0.12.8) were used to align reads to human genome GRCh37/hg19 with Ensembl 75 gene annotation. Only uniquely mapped reads were used to count reads aligned to each gene. The reads were quantified by htseq-count [98] (version 0.5.3p9) with Ensembl 75 gene sets. Gene differential expression analysis was performed using DESeq2 [58] (version 1.4.5). Genes which had no reads across all samples were discarded. Genes with and with more than a two-fold change in expression and an adjusted p-value of less than 0.1 were classified as significantly differentially expressed.

### Western blot analysis

Freshly isolated monocytes and MDM were lysed in radio immunoprecipitation assay buffer [150 mM NaCl, 50 mM Tris-Cl, pH 7.5, 1% Nonidet P-40, 0.5% sodium deoxicholate, 0.1% sodium dodecyl sulphate (SDS)] containing the complete protease inhibitor cocktail (Roche Molecular Biochemicals). Protein concentrations were determined by protein assay (Bio-Rad Laboratories). Cell lysates (10–20 μg per lane) were fractionated on 8-12% SDS-PAGE, electroblotted to nitrocellulose filters (Protran BA 85, Schleicher & Schuell), and probed with Abs anti-human A3A (rabbit polyclonal D23, Santa Cruz Biotechnology), anti-human A3G (goat polyclonal 109727, Abcam), anti-human SAMHD1 (rabbit polyclonal 366–380, Sigma-Aldrich), anti-human Mx2 (goat polyclonal N17, Santa Cruz Biotechnology) and anti-actin (mouse monoclonal Abs-5, BD Biosciences) as gel loading control. The anti p-SAMHD1 T592 Ab has been previously described [99]. SuperSignal West Femto Substrate (Pierce) and ECL Western blot detection Reagent (Amersham) were used according to the manufacturer’s instructions. Levels of A3A and Mx2 proteins were quantified using a GS-800 Calibrated Densitometer (Bio-Rad Laboratories).

### Statistical analysis

Data analysis was performed by using the Microsoft Office Excel 2007 software. Results are reported as means ± SE. Comparison between two groups was performed using paired, two-tailed t test. Values of p < 0.05 were considered statistically significant.

## Additional files

Additional file 1: Figure S1. MDM-derived CCL2 is effectively neutralized by anti-CCL2 Ab. MDM were treated with anti-CCL2 or control Ab (2.5 μg/ml) for 20 h and then infected with HIV-1_BaL_ (A) or (VSV-G) HIV-1 (B) as described in Methods. Supernatants were collected at 14 or 3 days post-infection, respectively, and the content of CCL2 was measured by ELISA. In A, data represent mean values (+SE) of the results obtained with 6 donors analyzed. In B, the results from 1 representative donor out of 2 tested are shown. Additional file 2: Figure S2. Endogenous CCL2 neutralization does not affect entry of HIV-1-based VLPs in MDM. MDM were treated with anti-CCL2 or control Ab (2.5 μg/ml) for 20 h and then challenged with VSV-G-VLP or Ada-VLP (1 μg of CAp24 equivalent per 10^5^ cells) as described in Methods. After 2 h, the percentage of GFP^+^ cells was assessed by flow cytometry. The flow cytometry histograms of 1 of the donors reported in Figure 2A are shown. Additional file 3: Figure S3. Endogenous CCL2 neutralization does not affect HIV-1 DNA intermediates synthesis. MDM were treated with anti-CCL2 or control Ab (2.5 μg/ml) for 20 h and then infected with HIV-1_BaL_ as described in Methods. Total DNA was extracted 24 h after infection and the levels of HIV-1 DNA intermediates (early, partial reverse and full-length transcripts) were assessed by semi-quantitative PCR (A) or quantitative Real Time PCR (B). The results from 1 representative donor out of 2 tested are shown. Additional file 4: Figure S4. Neutralization of endogenous CCL2 impacts the kinetic of HIV-1 DNA accumulation in MDM. MDM were treated with anti-CCL2 or control Ab (2.5 μg/ml) for 20 h and then infected with HIV-1_BaL_ as described in Methods. Total DNA was extracted 4 and 7 days after infection and the amount of total HIV-1 DNA (copies/10^6^ cells) was determined by qPCR. In A, the results from 1 representative donor out of 4 tested are shown. In B, data represent mean values (+SE) of the results obtained with all the donors analyzed. Additional file 5: Figure S5. Exogenous dNTPs supplementation increases the percentage of p24 Gag^+^ MDM. MDM were treated with anti-CCL2 or control Ab (2.5 μg/ml) for 20 h and then infected with HIV-1_BaL_ (3000 TCID_50_ per well) either in the absence or in the presence of dNTPs as described in Methods. After 14 days, cells were recovered and HIV-1 Gag expression was evaluated by flow cytometry. The results from 1 representative donor out of 4 tested are shown. Additional file 6: Figure S6. Neutralization of endogenous CCL2 does not induce A3G expression in MDM. MDM were treated with anti-CCL2 or control Ab (2.5 μg/ml) or IFN-β (1000 U/ml). After 20 h, cells were lysed and A3G protein expression in whole cell extracts was detected by western blot. Actin was used as house-keeping control. The result from one representative experiment of 3 independently performed is shown. Additional file 7: Figure S7. A3A expression is not affected by challenge with LV/Vpx in MDM. MDM were treated with anti-CCL2 or control Ab (2.5 μg/ml) for 20 h and then challenged or not with LV/Vpx as described in Methods. After 3 days, cells were lysed and A3A protein expression in whole cell extracts was detected by western blot. Actin was used as house-keeping control. The result from one representative donor out of 2 tested is shown. Additional file 8: Table S1. Differential expression analysis of genes modulated by CCL2 neutralization in MDM. Additional file 9: Table S2. Functional annotation clustering by DAVID of the 194 genes upregulated by CCL2 neutralization in MDM.
