# Erratum to: Endogenous CCL2 neutralization restricts HIV-1 replication in primary human macrophages by inhibiting viral DNA accumulation

**DOI:** 10.1186/s12977-015-0166-4

**Published:** 2015-06-09

**Authors:** Michela Sabbatucci, Daniela Angela Covino, Cristina Purificato, Alessandra Mallano, Maurizio Federico, Jing Lu, Arturo Ottavio Rinaldi, Matteo Pellegrini, Roberta Bona, Zuleika Michelini, Andrea Cara, Stefano Vella, Sandra Gessani, Mauro Andreotti, Laura Fantuzzi

**Affiliations:** Department of Hematology, Oncology and Molecular Medicine, Istituto Superiore di Sanità, Rome, Italy; Department of Therapeutic Research and Medicines Evaluation, Istituto Superiore di Sanità, Rome, Italy; National AIDS Center, Istituto Superiore di Sanità, Rome, Italy; Department of Molecular, Cell, and Developmental Biology, University of California Los Angeles, Los Angeles, California 90095 USA

After the publication of our article [1], we noted that Figure five (Figure 1 here) was published with part of the figure missing. We now provide a corrected version of Figure five (Figure 5) and wish to apologize for any inconvenience our error may have caused.

**Figure 1:**
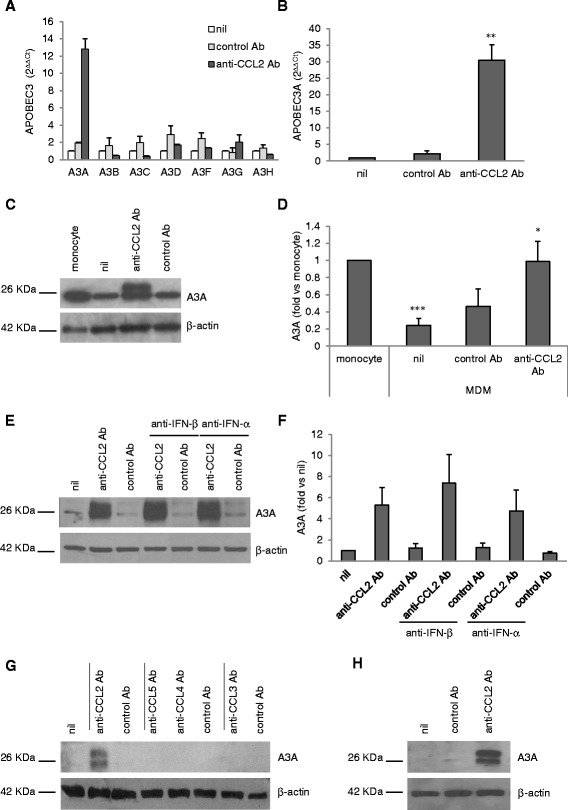
ᅟ
