# Supplementary figures and images for: Endogenous CCL2 neutralization restricts HIV-1 replication in primary human macrophages by inhibiting viral DNA accumulation
Source: Retrovirology. 2015 Jan 22;12:4. doi: 10.1186/s12977-014-0132-6 (PMC4314729; doi:10.1186/s12977-014-0132-6)

**A**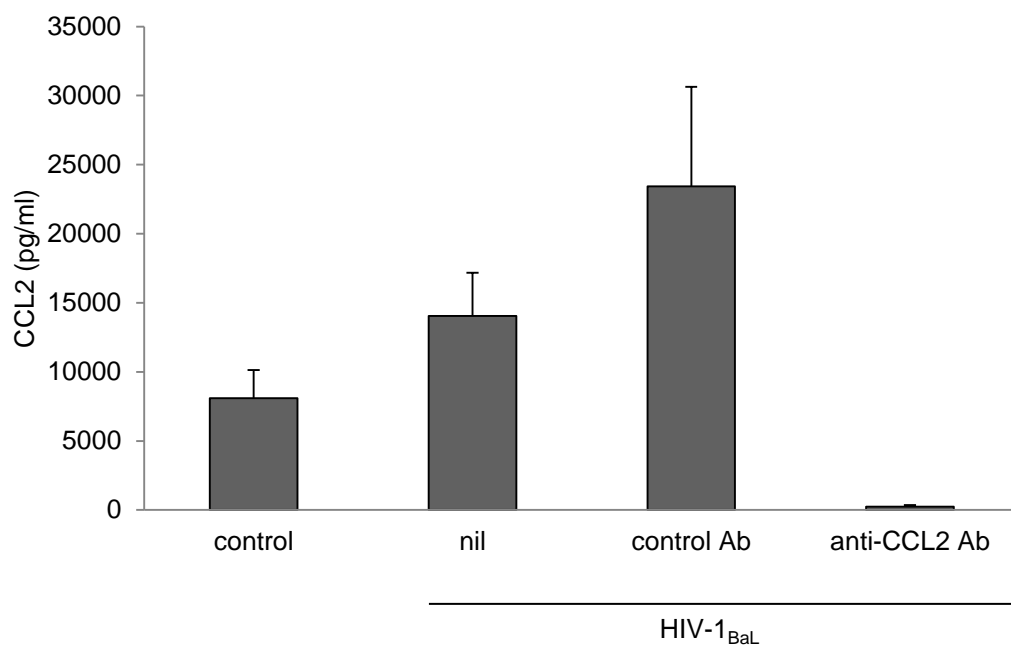**B**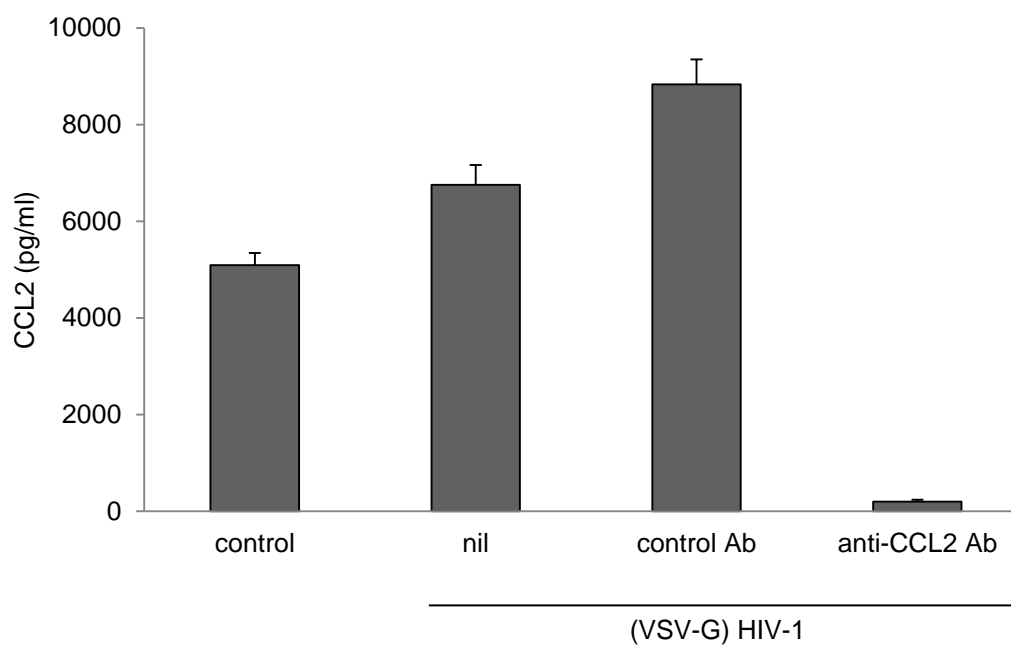

Supplement: Additional file 1: Figure S1. — MDM-derived CCL2 is effectively neutralized by anti-CCL2 Ab. MDM were treated with anti-CCL2 or control Ab (2.5 μg/ml) for 20 h and then infected with HIV-1BaL (A) or (VSV-G) HIV-1 (B) as described in Methods. Supernatants were collected at 14 or 3 days post-infection, respectively, and the content of CCL2 was measured by ELISA. In A, data represent mean values (+SE) of the results obtained with 6 donors analyzed. In B, the results from 1 representative donor out of 2 tested are shown. [file 12977_2014_132_MOESM1_ESM.pdf]

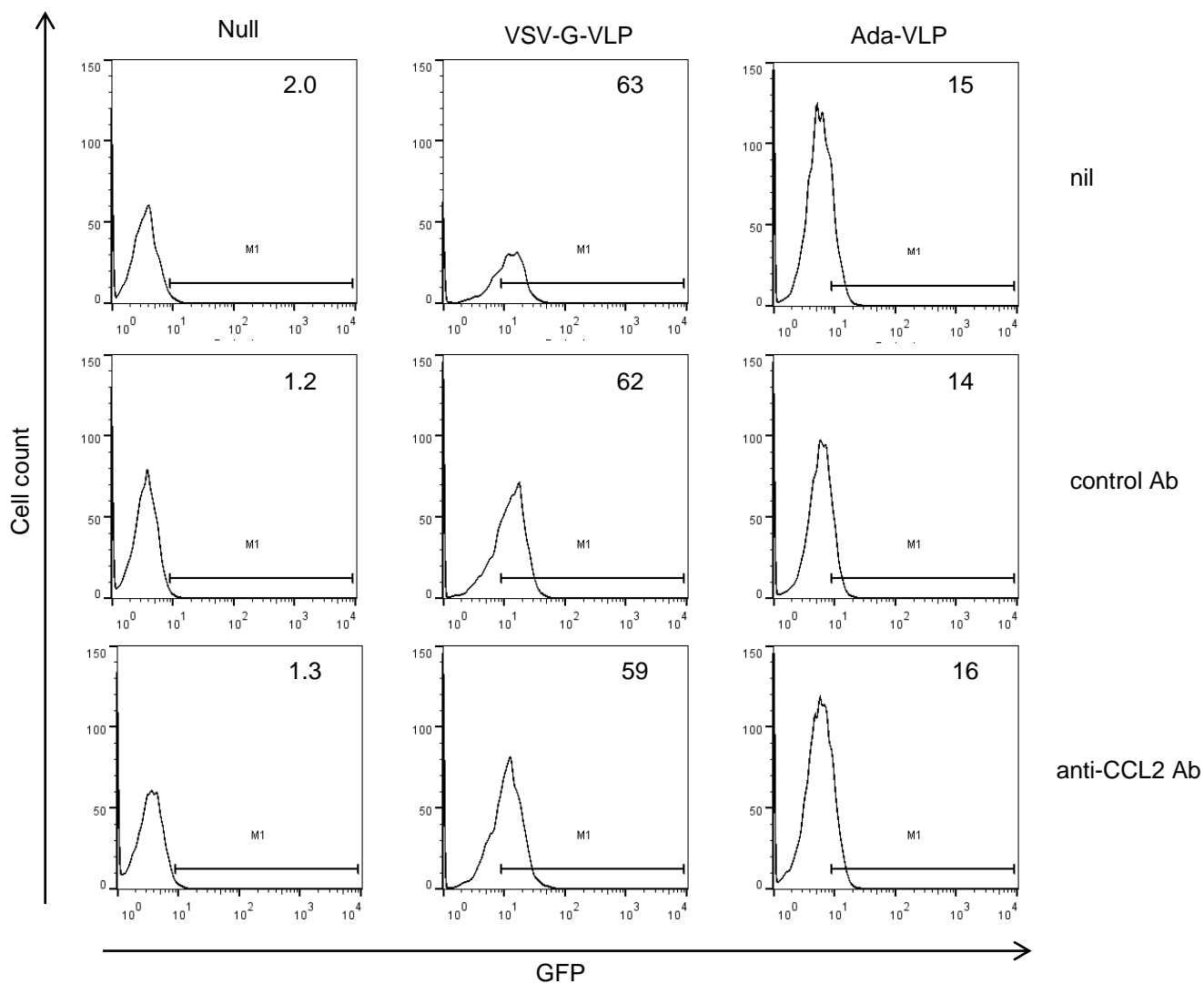

Supplement: Additional file 2: Figure S2. — Endogenous CCL2 neutralization does not affect entry of HIV-1-based VLPs in MDM. MDM were treated with anti-CCL2 or control Ab (2.5 μg/ml) for 20 h and then challenged with VSV-G-VLP or Ada-VLP (1 μg of CAp24 equivalent per 105 cells) as described in Methods. After 2 h, the percentage of GFP+ cells was assessed by flow cytometry. The flow cytometry histograms of 1 of the donors reported in Figure 2A are shown. [file 12977_2014_132_MOESM2_ESM.pdf]

A

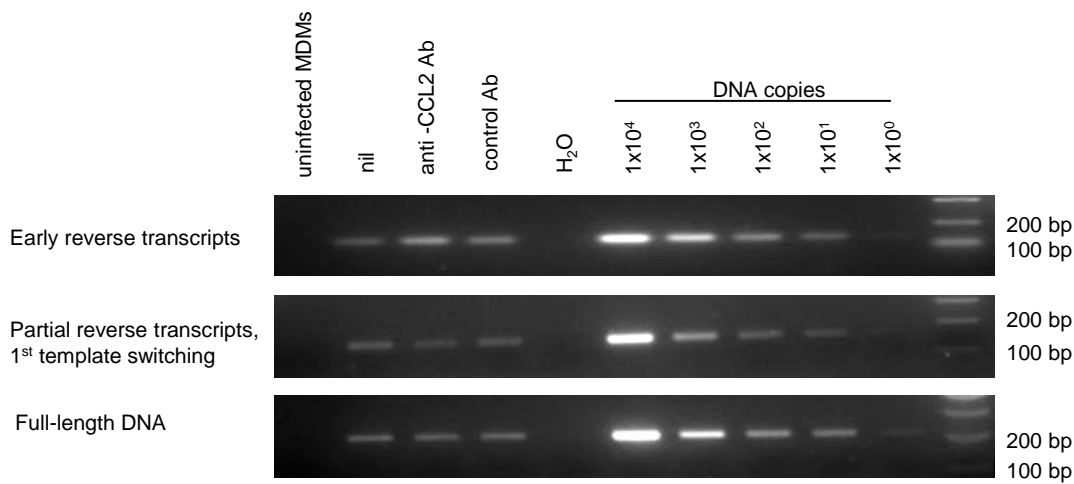

B

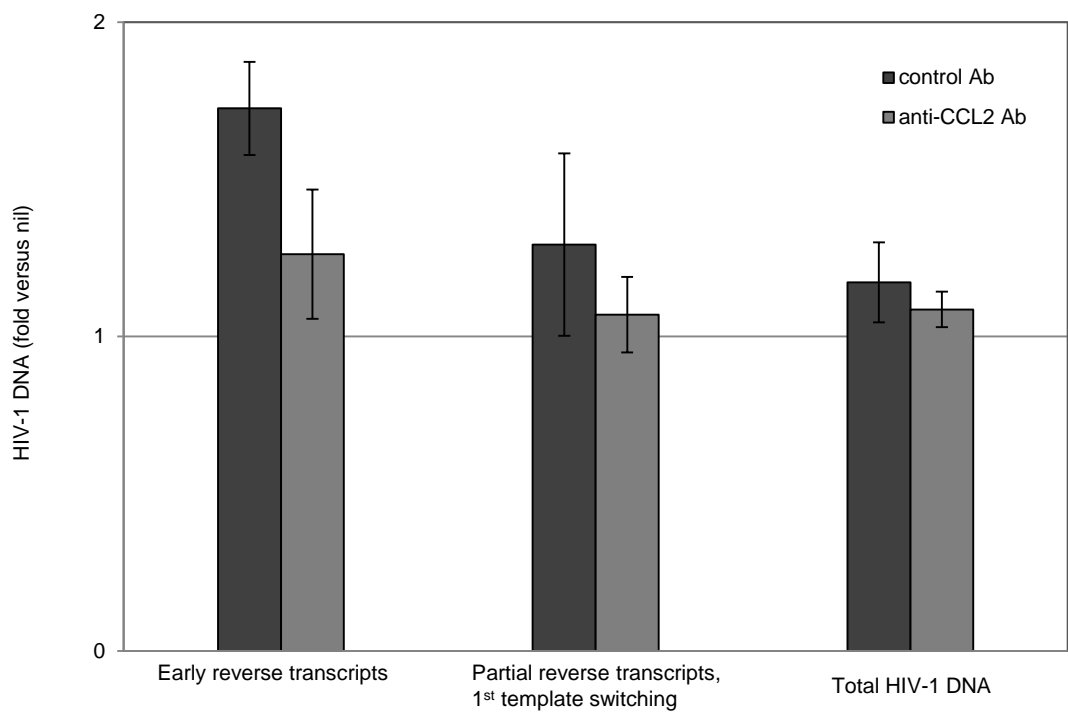

Supplement: Additional file 3: Figure S3. — Endogenous CCL2 neutralization does not affect HIV-1 DNA intermediates synthesis. MDM were treated with anti-CCL2 or control Ab (2.5 μg/ml) for 20 h and then infected with HIV-1BaL as described in Methods. Total DNA was extracted 24 h after infection and the levels of HIV-1 DNA intermediates (early, partial reverse and full-length transcripts) were assessed by semi-quantitative PCR (A) or quantitative Real Time PCR (B). The results from 1 representative donor out of 2 tested are shown. [file 12977_2014_132_MOESM3_ESM.pdf]

**A**

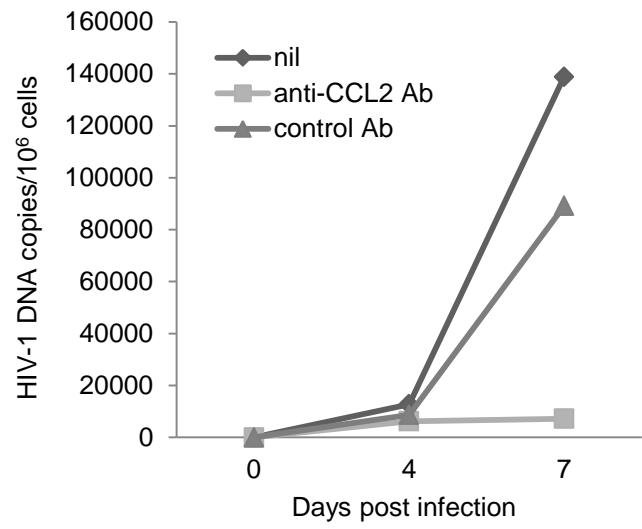

**B**

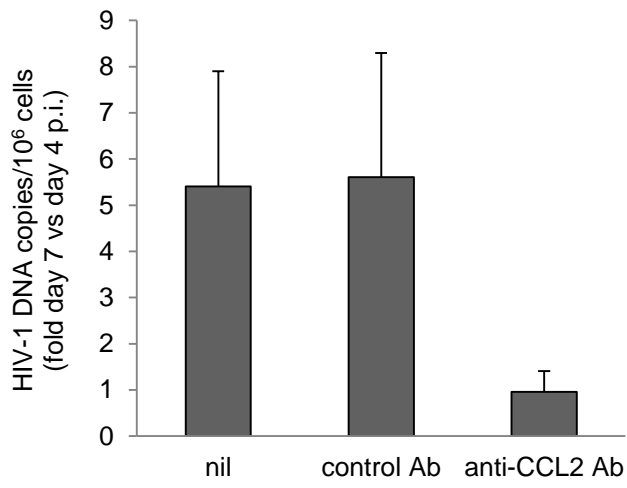

Supplement: Additional file 4: Figure S4. — Neutralization of endogenous CCL2 impacts the kinetic of HIV-1 DNA accumulation in MDM. MDM were treated with anti-CCL2 or control Ab (2.5 μg/ml) for 20 h and then infected with HIV-1BaL as described in Methods. Total DNA was extracted 4 and 7 days after infection and the amount of total HIV-1 DNA (copies/106 cells) was determined by qPCR. In A, the results from 1 representative donor out of 4 tested are shown. In B, data represent mean values (+SE) of the results obtained with all the donors analyzed. [file 12977_2014_132_MOESM4_ESM.pdf]

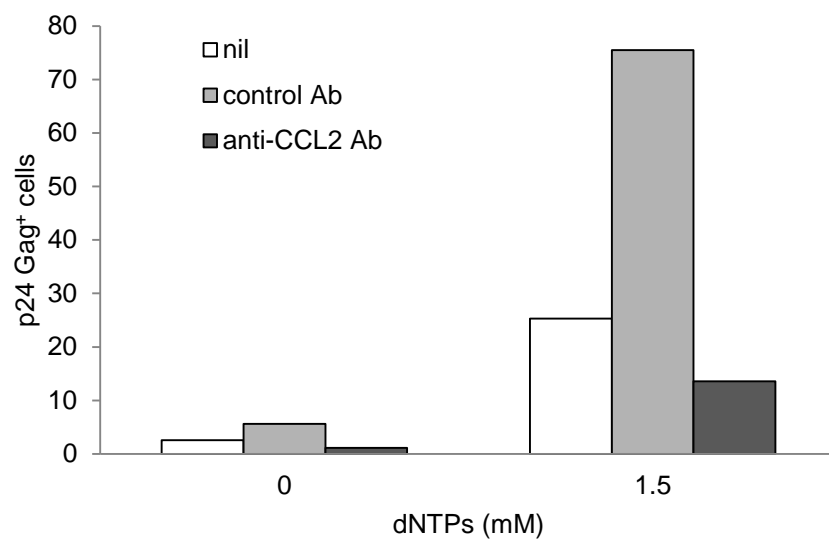

Supplement: Additional file 5: Figure S5. — Exogenous dNTPs supplementation increases the percentage of p24 Gag+ MDM. MDM were treated with anti-CCL2 or control Ab (2.5 μg/ml) for 20 h and then infected with HIV-1BaL (3000 TCID50 per well) either in the absence or in the presence of dNTPs as described in Methods. After 14 days, cells were recovered and HIV-1 Gag expression was evaluated by flow cytometry. The results from 1 representative donor out of 4 tested are shown. [file 12977_2014_132_MOESM5_ESM.pdf]

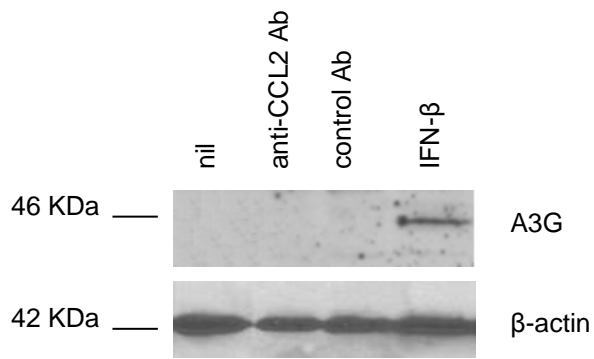

Supplement: Additional file 6: Figure S6. — Neutralization of endogenous CCL2 does not induce A3G expression in MDM. MDM were treated with anti-CCL2 or control Ab (2.5 μg/ml) or IFN-β (1000 U/ml). After 20 h, cells were lysed and A3G protein expression in whole cell extracts was detected by western blot. Actin was used as house-keeping control. The result from one representative experiment of 3 independently performed is shown. [file 12977_2014_132_MOESM6_ESM.pdf]

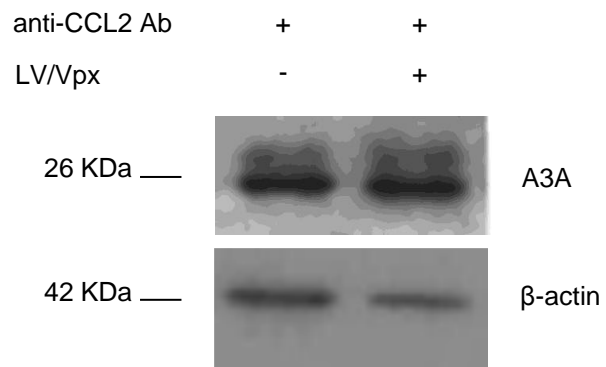

Supplement: Additional file 7: Figure S7. — A3A expression is not affected by challenge with LV/Vpx in MDM. MDM were treated with anti-CCL2 or control Ab (2.5 μg/ml) for 20 h and then challenged or not with LV/Vpx as described in Methods. After 3 days, cells were lysed and A3A protein expression in whole cell extracts was detected by western blot. Actin was used as house-keeping control. The result from one representative donor out of 2 tested is shown. [file 12977_2014_132_MOESM7_ESM.pdf]
